# Cardiac conduction system regeneration prevents arrhythmias after myocardial infarction

**DOI:** 10.1038/s44161-024-00586-x

**Published:** 2025-01-03

**Authors:** Judy R. Sayers, Hector Martinez-Navarro, Xin Sun, Carla de Villiers, Sarah Sigal, Michael Weinberger, Claudio Cortes Rodriguez, Leto Luana Riebel, Lucas Arantes Berg, Julia Camps, Neil Herring, Blanca Rodriguez, Tatjana Sauka-Spengler, Paul R. Riley

**Affiliations:** 1 Institute of Developmental and Regenerative Medicine, University of Oxford, Oxford, UK; 2 Department of Physiology, Anatomy and Genetics, University of Oxford, Oxford, UK; 3 Department of Computer Science, British Heart Foundation Centre of Research Excellence, University of Oxford, Oxford, UK; 4 Weatherall Institute of Molecular Medicine, University of Oxford, Oxford, UK; 5 Stowers Institute for Medical Research, Kansas City, MO USA

**Keywords:** Regeneration, Cardiac regeneration, Arrhythmias, Myocardial infarction

## Abstract

Arrhythmias are a hallmark of myocardial infarction (MI) and increase patient mortality. How insult to the cardiac conduction system causes arrhythmias following MI is poorly understood. Here, we demonstrate conduction system restoration during neonatal mouse heart regeneration versus pathological remodeling at non-regenerative stages. Tissue-cleared whole-organ imaging identified disorganized bundling of conduction fibers after MI and global His–Purkinje disruption. Single-cell RNA sequencing (scRNA-seq) revealed specific molecular changes to regenerate the conduction network versus aberrant electrical alterations during fibrotic repair. This manifested functionally as a transition from normal rhythm to pathological conduction delay beyond the regenerative window. Modeling in the infarcted human heart implicated the non-regenerative phenotype as causative for heart block, as observed in patients. These findings elucidate the mechanisms underpinning conduction system regeneration and reveal how MI-induced damage elicits clinical arrhythmogenesis.

## Abstract (web summary)

Sayers et al. reveal that heart regeneration during the neonatal period in mice extends to conduction system restoration after insult, highlighting the difference between this process and pathological remodeling in the adult heart.

## Main

The cardiac conduction system (CCS) is responsible both for initiation and conduction of the electrical impulses that bring about the estimated three billion rhythmic and synchronous contractions of the heart throughout the average human lifetime^1^. The electrical impulse is generated in the sinoatrial node and propagates sequentially through the atria, the atrioventricular node, the left and right bundle branches (BBs), the His–Purkinje network and finally through the ventricles. Following MI, disruption of normal heart rhythm is a major risk factor for elevated mortality. Ventricular tachycardia (characterized by an abnormally fast ventricular rate) and ventricular fibrillation (VF) (characterized by rapid and uncoordinated ventricular contractions) often present in both the early and chronic phases after MI (associated with reentrant behavior) and are a primary cause of sudden cardiac death. However, arrhythmias of the conduction system including atrioventricular block and left BB (LBB) block (LBBB) also arise during and after the transition from acute to chronic MI stages. Arrhythmia has largely been attributed to ischemic necrosis and scarring, albeit this does not explain the incidence of atrioventricular or BB heart block and dyssynchrony. How the CCS and particularly the fast-conducting BBs and Purkinje network of the ventricular conduction system (VCS)^2–6^ contribute mechanistically to arrhythmias remains unknown. More specifically, the extent to which His–Purkinje network cells die or remodel after MI and how the heart repairs the VCS are not well understood.

The adult mammalian heart lacks regenerative capacity and is unable to replace lost cells after injury. However, some neonatal mammals, including human infants, possess regenerative capacity for a short window after birth^7^. The neonatal mouse is capable of regenerating its heart, including replacement of lost cardiomyocytes and recovery of full function, following injury at postnatal day (P)1, but this ability is lost around day 4 after birth, with adoption of an adult-like fibrotic response by P7 (ref. ^8^). We hypothesized, therefore, that in response to MI, there would be cell loss or impairment of the CCS and specifically the VCS in non-regenerative P7 hearts but that the regenerative P1 heart would repair and fully restore the conduction system to maintain proper rhythm, with potential implications for the manifestation of clinical arrhythmias.

Here, we characterized the growth and expansion of the CCS over postnatal mouse development and compared the VCS injury responses of regenerative P1 hearts and non-regenerative P7 hearts using advanced whole-mount high-resolution imaging. We show that VCS cells are lost, and the bundling of VCS fibers is disrupted following MI in both P1 and P7 hearts. However, while the P1 heart reconstituted a functional His–Purkinje network, electrophysiological remodeling including downregulation of fast-conducting connexin 40 (CX40) and changes in localized ion channel expression persisted in P7 hearts. We identified the molecular differences underpinning these His–Purkinje network changes and demonstrated altered electrophysiological function from normal rhythm to pathological conduction delay across the regenerative window. Subsequent in silico modeling of the observed regional conduction loss in a human Purkinje network within a chronic MI heart determined the consequences of this non-regenerative electrical remodeling for arrhythmogenesis. These findings substantially enhance our understanding of the cellular and molecular changes in the VCS that drive clinically relevant electropathophysiological disturbances after MI.

## Results

### Postnatal development of the VCS

**Fig. 1:**
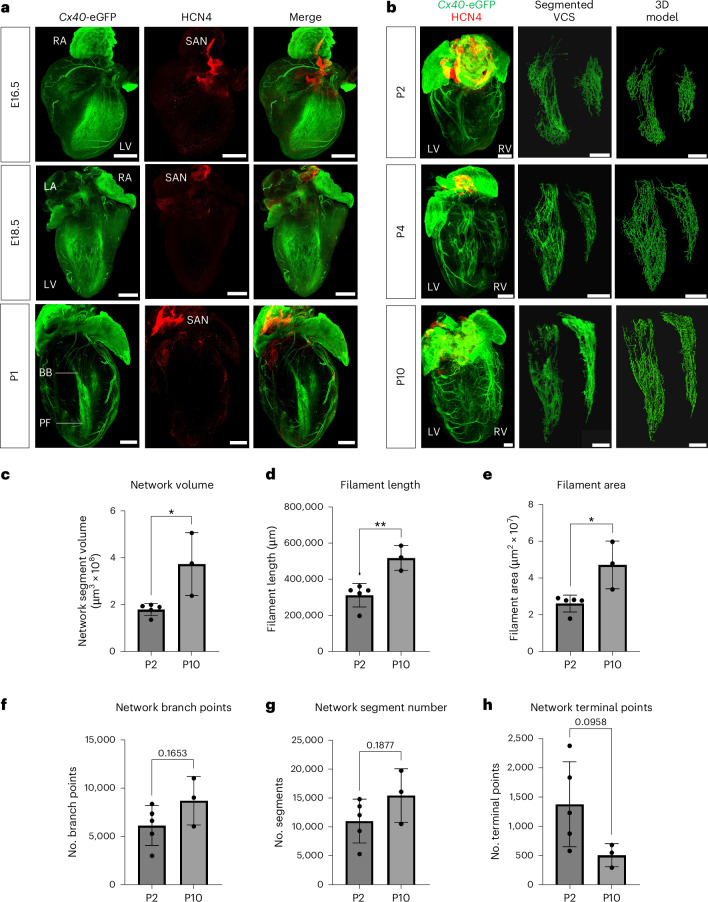
Developmental growth and expansion of the murine CCS. **a**, Three-dimensional-rendered tissue-cleared *Cx40*^eGFP/+^ E16.5 (top), E18.5 (middle) and P1 (bottom) hearts, revealing the refinement of trabecular CX40 expression to the developing His–Purkinje network. Green, *Cx40*-eGFP and anti-GFP antibody, labeling the developing VCS, atria and coronary arteries; red, HCN4, labeling nodal cells of the conduction system. **b**, Time series of tissue-cleared hearts through postnatal development from P2 to P10. Depicted from left to right, the 3D-rendered whole heart, the segmented VCS network and its 3D filament model. Scale bars, 500 μm. Labels indicate left atrium (LA), right atrium (RA), LV, right ventricle (RV), BBs, Purkinje fibers (PFs) and the sinoatrial node (SAN). **c**–**h**, Graphs quantifying network volume, filament length, filament area, number of branch points, number of segments and number of terminal points (that is, ends of branches) across postnatal development. Statistics: two-tailed unpaired *t*-test (*P < 0.05, **P < 0.01, ***P < 0.001); *P* = 0.0160 (**c**), *P* = 0.0054 (**d**), *P* = 0.0139 (**e**); mean values are depicted; error bars plot ±2 s.d.; *n* = 5 (P2) and *n* = 3 (P10) independent biological replicates. Source data

**Extended Data Fig. 1:**
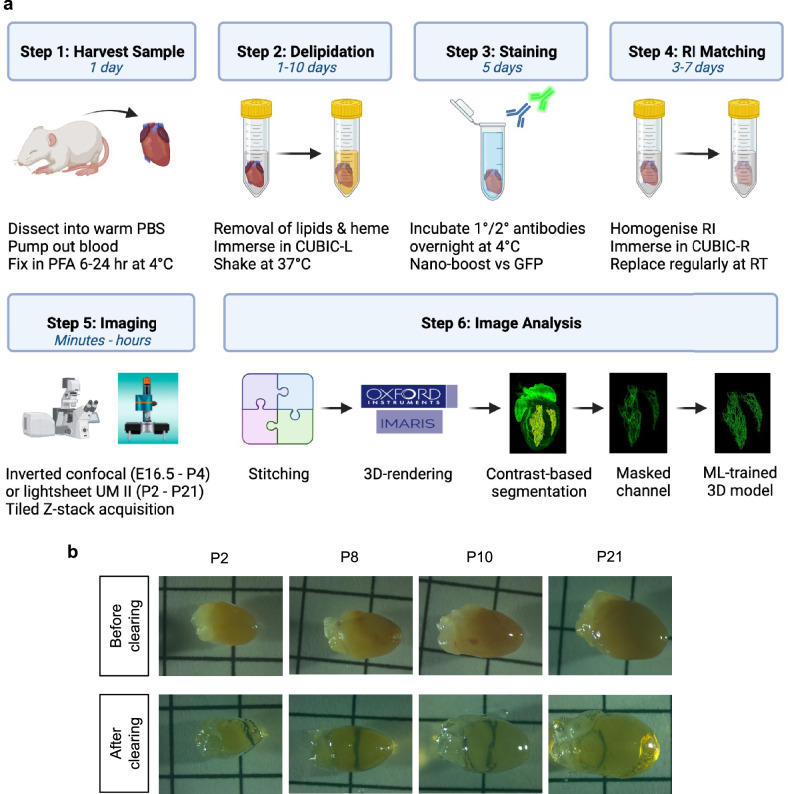
A pipeline for tissue clearing, imaging and analysis of mouse hearts through post-natal development. **a**. Schematic to illustrate the stages of the sample preparation, tissue clearing, immunostaining, imaging and analysis pipeline developed to enable wholemount imaging of the ventricular conduction system throughout post-natal mouse development. **b**. Time course of post-natal hearts imaged before and after processing through the tissue clearing pipeline. Scale: imaged on 5 mm graph paper. Schematic in **a** created with BioRender.com.

The VCS shares its developmental origins with working cardiomyocytes but becomes specialized during embryogenesis at around embryonic day (E)16.5 in the mouse through downregulation of the sarcomeric contractile apparatus and expression of specific ion channels and connexin isoforms^9–11^. We therefore set out to characterize late-stage formation and remodeling of the mouse VCS from E16.5 through postnatal development. Using a bespoke CUBIC (clear, unobstructed brain and/or body imaging cocktails and computational analysis) and immunostaining protocol to clear neonatal heart tissue while retaining endogenous green fluorescent protein (GFP) fluorescence (Extended Data Fig. 1), we visualized the intact neonatal VCS with the knockin *Gja5* (*Cx40*)-eGFP reporter line. We observed broad expression of CX40 across the trabecular myocardium at E16.5 through to restriction to the mature VCS structure by P1 (Fig. 1a). Following birth, substantial growth and expansion of the branching network continued through postnatal stages (Fig. 1b). Because CX40 is also expressed in the atria and coronary arteries, we segmented the VCS using machine learning to build a three-dimensional (3D) filament model of the VCS and quantified the network’s growth through postnatal development (Fig. 1c–h and Supplementary Videos 1–3). We identified a significant twofold increase in network volume between P2 and P10 (Fig. 1c), accompanied by a significant increase in filament length and total filament area (Fig. 1d,e). Despite expansion of the network during the postnatal period, the relative complexity was already established by P2 with minimal branching or increase in the number of network segments between P2 and P10 (Fig. 1f,g). Moreover, there was a trend toward a reduction in the number of terminal points during this period, suggesting that the fibers become more connected as the network matures (Fig. 1h). Interestingly, we found that expansion of the right ventricular network occurred later than that of the left, with more growth and increased complexity (Supplementary Fig. 1a,b). Overall, these findings indicate that the notable growth and maturation of the network after birth results from extension of existing Purkinje fibers, not from genesis of new branch points, and that there is more extensive development in the right than the left His–Purkinje network during postnatal stages.

### The His–Purkinje network is disrupted in injured P1 and P7 hearts

**Fig. 2:**
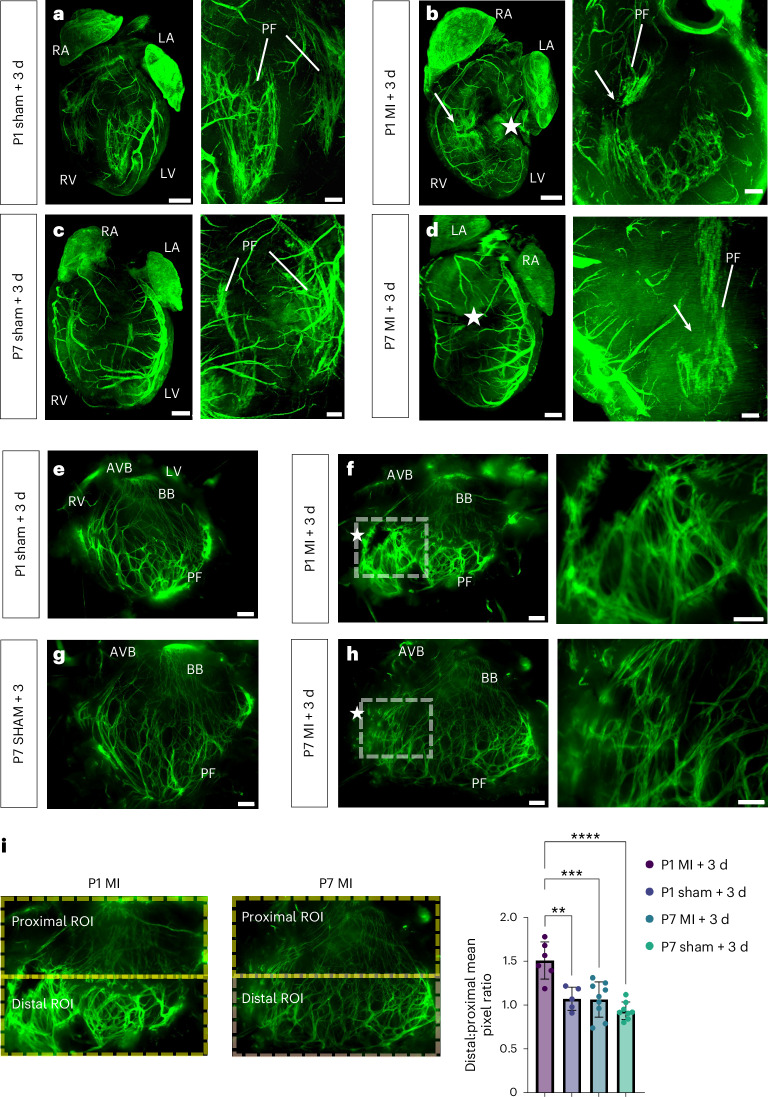
The His–Purkinje network is disrupted and the bundling of conduction fibers is altered by MI with differences in regenerative versus non-regenerative hearts. **a**–**d**, Three-dimensional-rendered tissue-cleared *Cx40*^eGFP/+^ hearts following MI or sham surgery at P1 (**a**,**b**) versus P7 (**c**,**d**), showing disruption of the His–Purkinje network after MI. Panel inserts show maximum intensity projections taken from the same heart, rotated by 90° to look side-on through the ventricle. Stars indicate the location of the LAD ligation. Arrows highlight regions of VCS disruption. Labels indicate left atrium, right atrium, left ventricle, right ventricle and Purkinje fibers. Scale bars, 500 μm for whole-heart images and 200 μm for magnified inserts. **e**–**h**, The dissected LV His–Purkinje network of *Cx40*^eGFP/+^ hearts 3 d after MI or sham surgery at the P1 (**e**,**f**) and P7 (**g**,**h**) stages. Panels show magnified inserts from the boxed regions of the corresponding P1 and P7 MI images, highlighting the disruption in fiber bundle morphology after MI. Stars indicate the level of the LAD ligation. Labels indicate the atrioventricular bundle (AVB), BBs and Purkinje fibers. Scale bars, 500 μm, 100 μm for magnified inserts. **i**, *Cx40*-eGFP signal is significantly increased in the distal Purkinje network below the level of the LAD ligation in P1 MI hearts 3 d after injury but not in P7 MI or P1 or P7 sham-operated hearts at an equivalent time point. ROI, region of interest. Statistics: one-way ANOVA with the Tukey–Kramer test for multiple comparisons (*P < 0.05, **P < 0.01, ***P < 0.001, ****P < 0.0001); *P* = 0.0015 (P1 MI + 3 d versus P1 sham + 3 d), *P* = 0.002 (P1 MI + 3 d versus P7 MI + 3 d), *P* < 0.0001 (P1 MI + 3 d versus P7 sham + 3 d); mean values are depicted; error bars plot ±2 s.d.; *n* = 6 (P1 MI + 3 d), *n* = 5 (P1 sham + 3 d), *n* = 9 (P7 MI + 3 d), *n* = 8 (P7 sham + 3 d) independent biological replicates. Source data

Next, we investigated whether there were gross morphological and cellular changes to the His–Purkinje network following MI induced by left anterior descending (LAD) coronary artery ligation. We first analyzed the network structure at 3 d after MI of *Cx40*^eGFP/+^ regenerative P1 and non-regenerative P7 mice (Fig. 2). *Cx40*-eGFP^+^ fibers within the network below the level of the suture site were discontinuous as compared to sham surgical controls, indicating that VCS cells were either lost or molecularly altered after MI (Fig. 2a–d). Three-dimensional reconstruction of MI hearts suggested that loss of GFP expression proximal to the suture site resulted from tissue damage to the VCS cells downstream of the MI event (Fig. 2b,d, right).

To assess changes in bundling and fiber morphology after MI, we dissected open the left ventricle (LV) of hearts at 3 d after MI to expose the His–Purkinje network (Fig. 2e–h and Supplementary Fig. 2). In both P1 and P7 infarcted hearts, gaps were evident in the His–Purkinje network proximal to the LAD ligation site, and there were bright CX40-expressing bundles with altered morphology proximal to the injury region evident in P1 hearts (Fig. 2h, right). However, at P1, there was greatly increased overall *Cx40*-eGFP expression in the distal half of the network below the level of the suture site (Fig. 2i), which was not seen after MI at P7. In addition, at P7, there was reduced fiber bundle formation and a disorganized network (Fig. 2h, right). This is important, as there is a close link between bundle anatomy and VCS function^12^: bundling of VCS cells within the fibrous sheath affects electrical insulation from the working myocardium, partially in the mouse or fully in large mammals including humans, to ensure fast electrical propagation through the network^13–15^.

### Distinct VCS transcriptional signatures across the regenerative window

We next determined whether there was a loss of specific subsets of conduction cells within the network and/or changes in the molecular response to injury across the regenerative window.

**Fig. 3:**
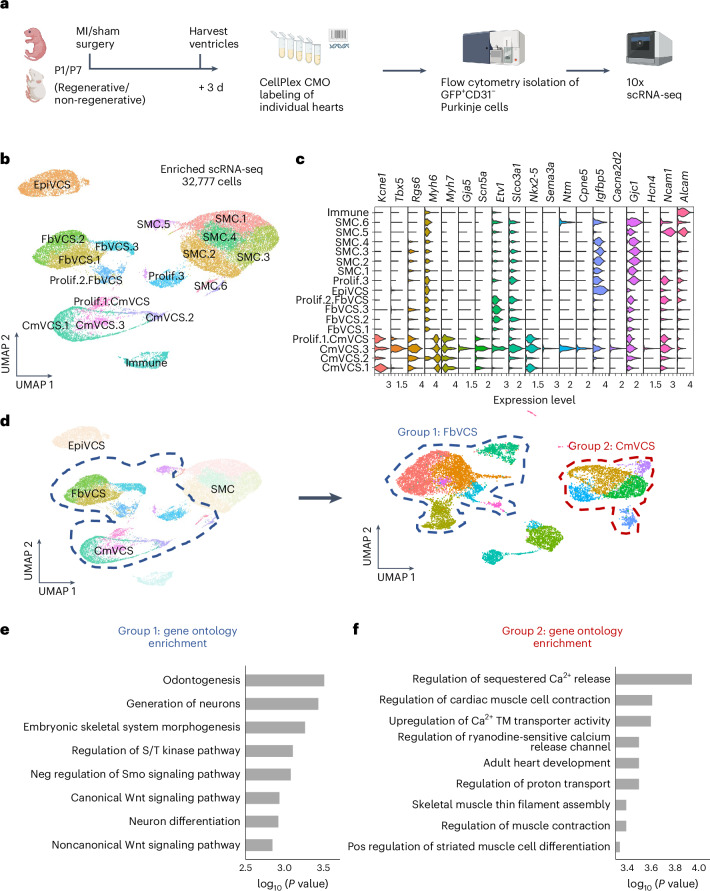
scRNA-seq identifies a heterogeneous transcriptomic signature across the VCS of injured and regenerating murine hearts. **a**, Schematic to illustrate the experimental design, including neonatal mouse surgeries, the 3-d-after-injury harvest time point, the CellPlex multiplexing strategy, flow cytometry enrichment and 10x scRNA-seq. CMO, cell multiplexing oligo. **b**, Uniform manifold approximation and projection (UMAP) visualization of cells integrated across all conditions reveals substantial heterogeneity across the dataset. Cm, cardiomyocyte like; epi, epicardial like; Fb, fibroblast like; SMC, smooth muscle cell. **c**, Violin plot showing expression of known conduction system markers across clusters in the dataset. The core Purkinje cluster CmVCS.3 shows particularly conserved expression of these canonical conduction system markers. **d**, Reclustering following removal of smooth muscle, immune and epicardial-like cell populations. Reclustered UMAP identifies two major groups of fibroblast-like (group 1, FbVCS) and cardiomyocyte-like (group 2, CmVCS) conduction cells. **e**, Top gene ontology enrichment terms for genes upregulated in group 1 fibroblast-like VCS cells (Fisher’s exact test). Neg, negative. **f**, Top gene ontology enrichment terms for genes upregulated in group 2 cardiomyocyte-like VCS cells (Fisher’s exact test). Pos, positive; TM, transmembrane. Schematic in **a** created with BioRender.com.

Because the VCS shares its developmental origins with working cardiomyocytes, we reanalyzed a published cardiomyocyte-enriched single-nuclear RNA-sequencing dataset from P1 and P8 hearts 1 and 3 d after MI or sham surgery^16^ to extract VCS cells computationally from within the dataset (Supplementary Fig. 3). Following reclustering, a single cluster (cluster 13) contained known VCS markers among its five most highly conserved genes, which included *Etv1* (ref. ^17^) (ETS transcription factor) and *Slco3a1* (the CCS *lacZ* locus)^18^. The other main VCS marker genes identified from an existing (E16.5) published VCS-enriched scRNA-seq dataset^19^ were also upregulated in cluster 13 (Supplementary Fig. 3b). Assigning a VCS gene module score based on expression of seven canonical VCS markers revealed higher relative expression in cluster 13 than in other cardiomyocyte clusters, indicating that this is indeed a VCS population (Supplementary Fig. 3c). Differential expression analysis between cluster 13 and the rest of the cardiomyocytes identified both known and new genes specific to the VCS including *Etv1*, *Gja5* (CX40), *Kcnj3* (inwardly rectifying potassium channel member) (Supplementary Fig. 3d, known); *Grip1* (neuronal scaffold protein), *Meg3* (long noncoding RNA) and *Vcan* (versican, extracellular matrix component) (Supplementary Fig. 3e, new). However, the low number of VCS cells captured in cluster 13 from the snRNA-seq P1 versus P8 dataset provided limited power for analyzing Purkinje-specific cellular responses to MI. We therefore performed scRNA-seq on injured P1 and P7 hearts and enriched for VCS cells by fluorescence-activated cell sorting for *Cx40*-eGFP-positive cells to determine the transcriptional signature of the regenerating VCS (Fig. 3a). Given that CX40 is also expressed in the atria and coronary arteries, we dissected off the atria and excluded endothelial cells using negative selection for CD31 (Supplementary Fig. 4). Hearts were harvested for sequencing 3 d after MI or sham surgery to coincide with clear morphological changes in the VCS (Fig. 2). Furthermore, the time point of 3 d after MI captures relatively early molecular changes, concomitant with the onset of the cardiomyocyte regenerative response^16^, that might define longer-term VCS restoration or remodeling. We optimized a gentle mechanical–chemical dissociation and enrichment protocol to sort a high number of viable *Cx40*-eGFP^+^ cells from individual hearts and by performing lipid labeling of the single-cell suspensions and subsequent demultiplexing determined that there were no major differences in cell type composition across different hearts and with extent of injury, either within the same time point or treatment group (Supplementary Figs. 5 and 6).

**Extended Data Fig. 2:**
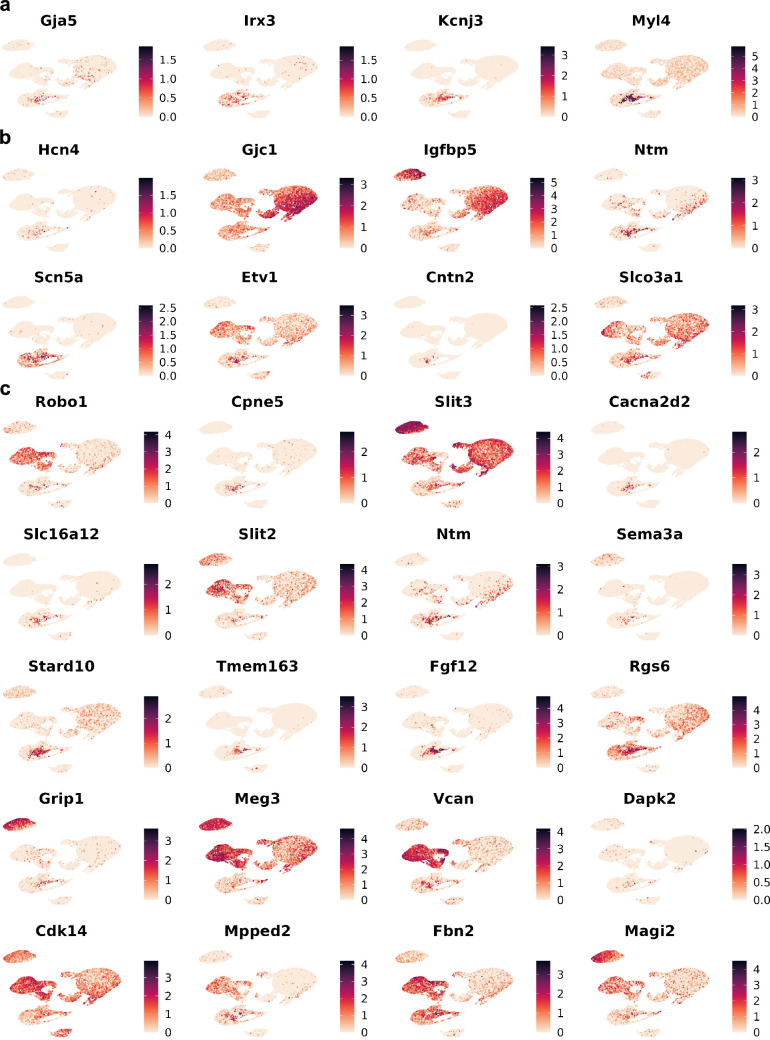
Expression of known ventricular conduction system markers show heterogeneous expression across the enriched CX40 + CD31- single-cell RNA-sequencing dataset. **a**. Featureplots of *Gja5*, *Irx3*, *Kcnj3* and *Myl4* identify a core Purkinje cluster with most highly conserved expression of ventricular conduction system (VCS) markers. **b**. Featureplots of other well-known VCS markers show varied expression patterns across the CX40 + CD31- single-cell RNA-sequencing dataset. **c**. Featureplots of the most differentially expressed genes of the conductive VCS cluster 13 isolated from the cardiomyocyte-enriched single-nuclear RNA-sequencing dataset^1^ also show varied expression patterns across the CX40 + CD31- single-cell RNA-sequencing dataset.

Clustering revealed striking heterogeneity across the VCS with two major separate clusters, based on known VCS markers, each showing either more cardiomyocyte-like or fibroblast-like expression patterns. In addition, we identified an epicardial-like population, a small immune population and a smooth muscle cell population resulting from CX40 expression in smooth muscle cells of the coronary arteries (Fig. 3b and Supplementary Fig. 7a). We identified a core cluster with conserved expression of canonical Purkinje genes, CmVCS.3 (Fig. 3c and Extended Data Fig. 2). To interrogate the differences between the VCS clusters, we integrated our scRNA-seq data with a stage-matched whole-heart (minus cardiomyocytes) scRNA-seq reference dataset from hearts 3 d following MI or sham injury^20^ and found that, after anchor selection, while the cardiomyocyte-like VCS cells clustered separately from the reference, the epicardial-like and fibroblast-like populations integrated with epicardial and fibroblast clusters, respectively (Supplementary Fig. 8). We then performed reclustering after removing the epicardial-like cells and the immune and smooth muscle cell clusters to elucidate the specific differences between the cardiomyocyte-like versus fibroblast-like conduction populations (Fig. 3c and Extended Data Fig. 2). We found enrichment of gene ontology terms associated with neuronal differentiation and Wnt–Smoothened signaling pathways in the fibroblast-like VCS cluster grouping 1 versus control of calcium ion release and contraction-associated gene sets in the cardiomyocyte-like VCS grouping 2 (Fig. 3d,e and Supplementary Fig. 7b). We suggest, therefore, that fibroblast-like VCS cells represent a previously uncharacterized conduction-associated cell type, showing similarity at the level of gene expression to cardiac fibroblasts but with an additional conductive signature including synaptic, neuronal, cell–cell adhesion and ion channel gene sets.

### Repopulation of VCS cells during regeneration

**Fig. 4:**
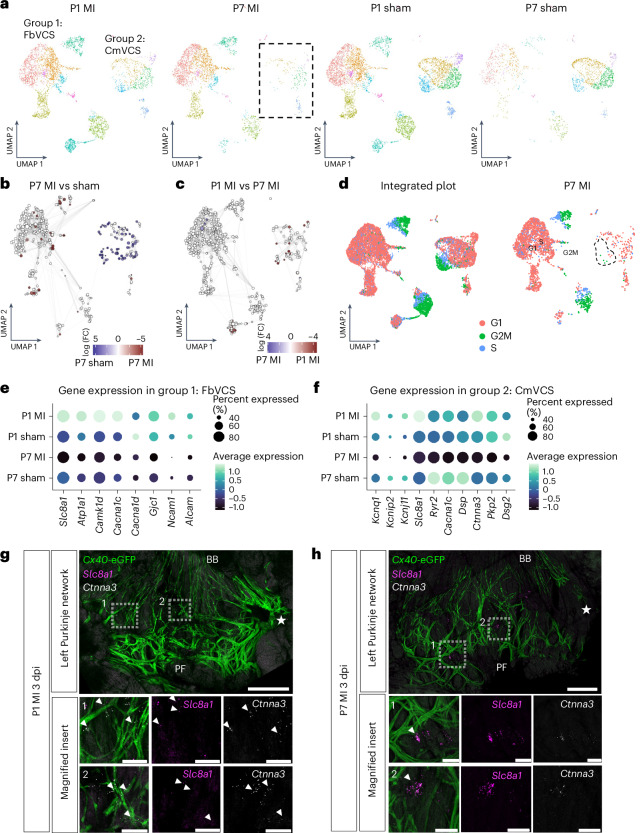
Shift in VCS composition and electrical remodeling of conductive subpopulations following MI in regenerating versus non-regenerating hearts. **a**, UMAP visualization of the reclustered scRNA-seq data following removal of smooth muscle, immune and epicardial-like VCS cell populations, split by age (P1 versus P7) and treatment (MI versus sham). The boxed region highlights a relative decrease in the group 2 cardiomyocyte-like VCS cluster in the P7 MI dataset. **b**,**c**, Milo differential abundance testing identifies significantly enriched or reduced neighborhoods between non-regenerative P7 sham-operated versus MI hearts (**b**) and infarcted regenerative P1 versus non-regenerative P7 hearts (**c**). **d**, Cell cycle analysis identifies three cycling populations in the reclustered scRNA-seq dataset. The cardiomyocyte-like VCS cycling population is missing in the P7 MI dataset, as highlighted by the dashed region. **e**, Dot plot showing average expression of ion channel and cell–cell adhesion markers with significant differential expression between P1 versus P7 MI hearts within the fibroblast-like VCS group 1. These markers were all upregulated in P1 MI hearts but downregulated in the P7 MI heart. **f**, Dot plot showing average expression of ion channel and cell–cell adhesion markers with significant differential expression between P1 versus P7 MI hearts within the cardiomyocyte-like VCS group 2. These markers were all upregulated in P1 MI hearts but downregulated in the P7 MI heart. In **e**,**f**, only genes not differentially expressed between the P1 sham and P7 sham datasets (using a log_2_ (FC) cutoff threshold of 0.1) were analyzed to remove developmentally driven expression changes. **g**,**h**, HCR gene expression analysis of *Slc8a1* (magenta) encoding the NCX1 sodium–calcium exchanger and *Ctnna3* (gray) encoding α-catenin on the dissected LV His–Purkinje network of P1 (**g**) and P7 (**h**) hearts 3 d after MI (dpi). Numbered inserts below the main image show magnified panels from the corresponding boxed regions. Arrowheads in **g** highlight HCR probe expression overlapping with *Cx40*-eGFP-positive conduction fibers in the P1 heart. Arrowheads in **h** highlight HCR probe expression in *Cx40*-eGFP-negative gaps in the conductive network. The experiment was repeated independently three times with similar results. Stars indicate the level of the LAD ligation. Labels indicate BBs and Purkinje fibers. Scale bars, 500 μm for whole-heart images and 100 μm for magnified inserts.

**Extended Data Fig. 3:**
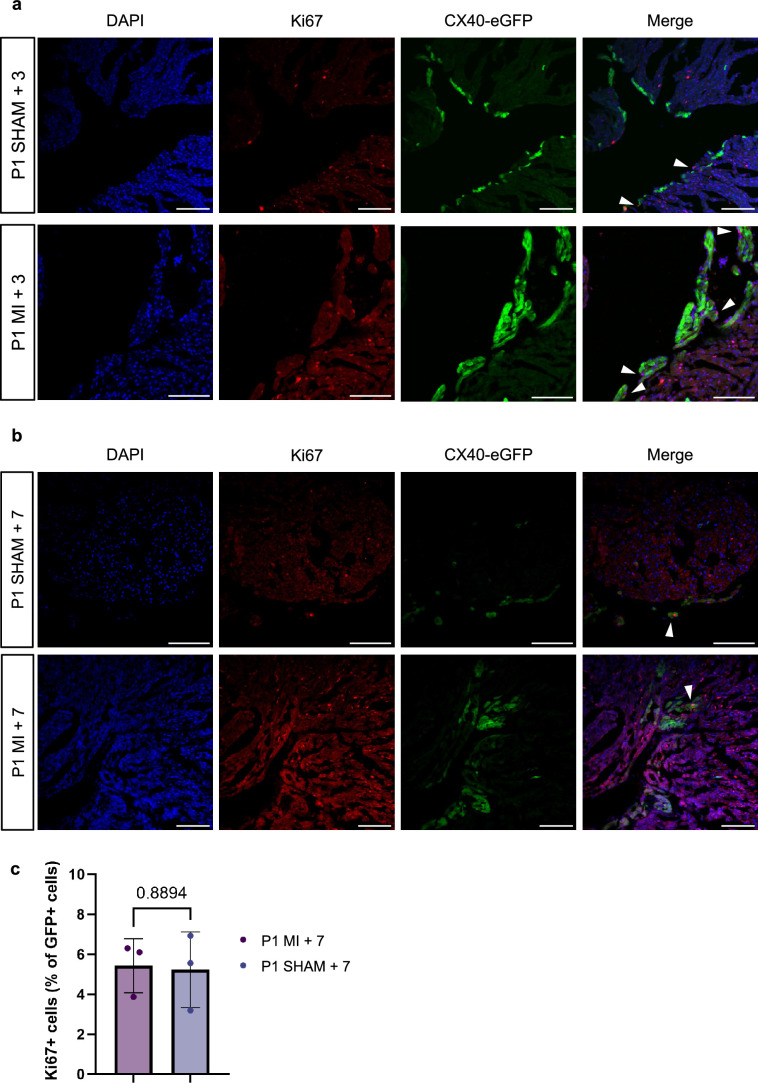
Enhanced proliferation of pre-existing CX40-eGFP+ cells does not drive regeneration of the ventricular conduction system. **a**. Ki67+ cycling cells are commonly found in non-conductive cells adjacent to the CX40-eGFP-positive VCS in P1 hearts that underwent sham or MI surgery, 3 days after injury. White arrows indicate Ki67 + CX40-eGFP-negative cells. **b**. There are a small number of cycling VCS cells in the P1 heart 7 days following either sham or MI surgery. White arrows indicate rare Ki67 + Cx40-eGFP-positive cells. Scale bars: 100μm. **c**. Quantification of the proportion of GFP-positive cells that are also Ki67+ in P1 hearts, 7 days following MI or sham treatments. Statistics: Two-tailed unpaired t test; error bars plotted for +/− 2 SD; n = 3 independent biological replicates Source data

Next, we interrogated differences in VCS cell type composition between MI versus sham and regenerative versus non-regenerative conditions in the reclustered dataset (Fig. 4). While cell clusters were more equally represented across P1 MI and sham datasets, there was a notable change in VCS cell composition in P7 MI hearts: a major shift in cellular representation away from the more cardiomyocyte-like (group 2) VCS cells in P7 sham-operated hearts to the more fibroblast-like (group 1) VCS cells in P7 MI hearts (Fig. 4a). By assigning cells to neighborhoods on a k-nearest-neighbor graph^21^, we found that almost all the local neighborhoods containing group 2 cardiomyocyte-like VCS cells were significantly reduced in P7 MI versus sham-operated hearts (Fig. 4b) but retained in infarcted P1 hearts, including those containing the core Purkinje cells with the most highly conserved VCS marker expression (Fig. 4c). Analysis of cell cycle markers identified a cycling VCS group 2 population that was lost in P7 MI hearts as compared to sham-operated or P1 MI hearts (Fig. 4d). However, proliferation of VCS cells following MI is unlikely to be a major determinant of the regenerative response, because the size of the cycling group 2 population was relatively small. This was confirmed by immunofluorescence staining, which did not show a significant increase in the proportion of *Cx40*-eGFP^+^ VCS cells positive for the mitotic marker Ki-67 between P1 hearts following MI or sham surgery (Extended Data Fig. 3).

**Extended Data Fig. 4:**
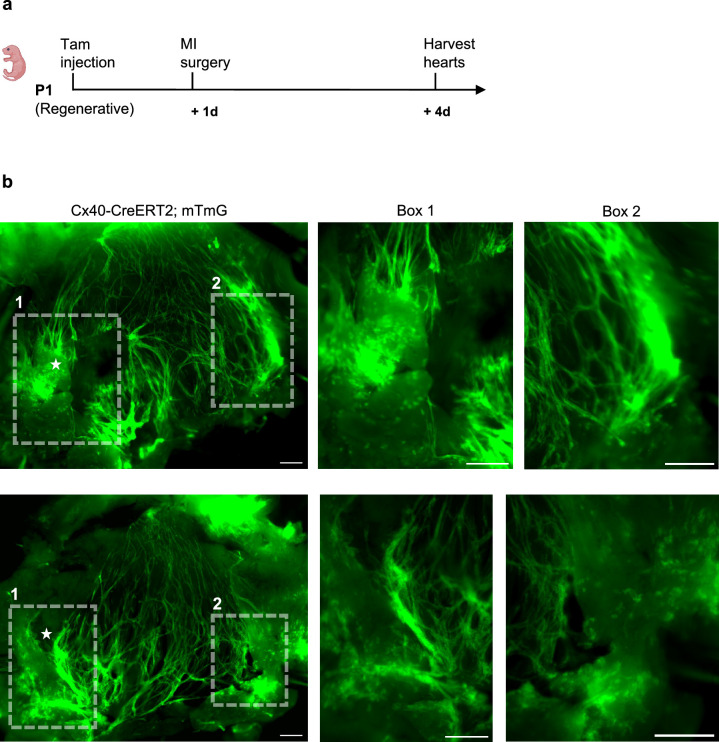
Existing Cx40-positive cells concentrate around damaged areas of the His/Purkinje network during regeneration. **a**. Schematic to illustrate experimental design. Cx40-CreERT2/+; mTmG pups were injected with tamoxifen on P1. MI surgery was performed on P2, and hearts were then harvested 4 days later for VCS dissection. **b**. The dissected *CreERT2; mTmG* VCS of hearts dissected 4dpi, imaged under green fluorescence (488 nm). Numbered boxes on the right-hand side show magnified panels from the corresponding boxed regions, in which concentrations of GFP-positive traced cells are found adjacent to the injured area of the network. Stars indicate location of the LAD ligation. Scale bars: 500μm, 100μm for zoomed inserts. Schematic in **a** created with BioRender.com.

Given that no significant differences were identified in VCS cell type composition between P1 MI versus sham-operated hearts, we next sought to identify the source of VCS repopulation during regeneration. To determine whether regeneration occurs through repopulation of the Purkinje network by existing *Cx40*^+^ cells, we performed lineage-tracing experiments using an inducible *Cx40-*Cre^ERT2^ line, providing tamoxifen at P1 followed by MI surgery at P2. We found that by 4 d after injury, on dissection of the VCS, the main network had continuous GFP-positive signal, as expected, but additionally, there were evident concentrations of *Cx40*^+^-traced cells adjacent to the area of VCS damage that were not yet integrated into the network (Extended Data Fig. 4). These results suggest that regeneration of the VCS network involves existing CX40^+^ cells, which likely migrate to the injury area and subsequently integrate into the network to replace lost His–Purkinje cells.

### Loss of electrical identity and ionic remodeling of the VCS after injury

**Extended Data Fig. 5:**
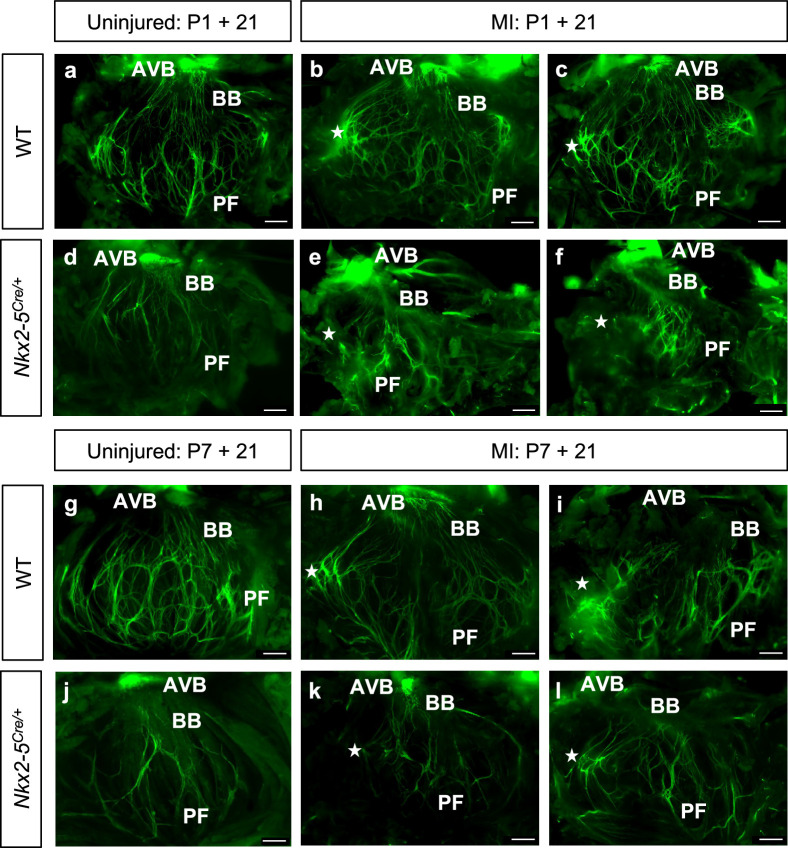
Nkx2-5 regulates regeneration of the His/Purkinje network after myocardial infarction. **a–c**. Hearts that underwent MI surgery at P1, 21 days after injury (b-c) together with the stage-matched uninjured control (a). Overall regeneration of the His/Purkinje network is seen following MI at P1. **d–f**. Hearts from *Nkx2-5*^*Cre/+*^ haploinsufficient animals with a hypomorphic conduction system, that underwent MI surgery at P1, 21 days after injury (e-f) together with the stage-matched uninjured control (d). The reduced levels of Nkx2-5 lead to very disrupted His/Purkinje network morphology as compared to the uninjured hypomorphic control. In all cases each image shows a separate individual’s heart. **g–i**. Hearts that underwent MI surgery at P7, 21 days after injury (h-i) together with the stage-matched uninjured control (g). The His/Purkinje network has sustained alterations following MI at P7. **j–l**. Hearts from *Nkx2-5*^*Cre/+*^ haploinsufficient animals with a hypomorphic conduction system, that underwent MI surgery at P7, 21 days after injury (k-l) together with the stage-matched uninjured control (j). The hearts recover after P7 MI to a similar extent to the wild-type hearts despite reduced dosage of Nkx2-5. Stars indicate location of the LAD ligation. Labels indicate atrioventricular bundle (AVB), bundle branches (BB), Purkinje fibres (PF). Scale bars: 500μm

We further analyzed differential gene expression in the VCS between P1 versus P7 hearts. Focusing on the core Purkinje cluster with the most highly conserved expression of VCS marker genes, CmVCS.3 (Supplementary Fig. 9a), and on the proliferating VCS cluster significantly reduced in P7 MI hearts, prolif.1.CmVCS (Supplementary Fig. 9b), we identified a cohort of genes upregulated in the VCS of infarcted P1 hearts that were downregulated in infarcted P7 hearts. These genes generally fall into at least one of three categories: regulators of gene transcription (for example, *Nkx2-5* (NK2 homeobox 5), *Cdk8* (cyclin-dependent kinase 8), *Hmgn2* (high-mobility group nucleosomal binding domain 2), *Prdm16* (PR domain-containing 16)), implicated in arrhythmia in genome-wide association or knockout studies (for example, *Atxn1* (ataxin 1), *Mical2* (microtubule-associated monooxygenase, calponin- and LIM-containing 2), *Ppargc1b* (peroxisome proliferative-activated receptor γ coactivator 1β), *Fstl1* (follistatin-like 1)) and/or intracellular calcium mobilization or synaptic signaling (for example, *Pde4d* (phosphoesterase 4D, cAMP specific), *Inpp5a* (inositol polyphosphate-5-phosphatase A), *Jph2* (junctophilin 2), *Gphn* (gephyrin)). While these genes showed significantly reduced expression in infarcted P7 hearts as compared to infarcted P1 hearts, we sought to analyze injury-specific rather than developmental gene expression changes and focused on genes in this list for which there was no significant difference in expression between P1 and P7 sham-operated hearts, taking a cutoff threshold of average log_2_ (fold change (FC)) = 0.1. In agreement with the scRNA-seq expression analysis, hybridization chain reaction (HCR) analyses found that, while *Pde4d* (scRNA-seq P1 MI versus P7 MI adjusted *P* value, 4.914 × 10^−6^; average log_2_ (FC), 0.251) and *Nkx2-5* (scRNA-seq P1 MI versus P7 MI adjusted *P* value, 1.119 × 10^−7^; average log_2_ (FC), 0.383) were upregulated in *Cx40*-eGFP^+^ Purkinje fibers in infarcted P1 hearts, there was very low expression in the infarcted P7 network (Supplementary Fig. 9c,d). *Nkx2-5* has previously been implicated in maintenance of the conductive phenotype in the VCS^22^. To validate the functional role of *Nkx2-5* in the regenerating VCS, we performed MI and sham surgeries on *Nkx2-5*^Cre/+^ haploinsufficient neonates with a hypomorphic conduction system. While repair in non-regenerative P7 *Nkx2-5*^Cre/+^ heterozygotes following MI seemed comparable to that of wild-type *Nkx2-5*^+/+^ littermates, P1 heterozygotes had severely reduced ability to regenerate a normal network (Extended Data Fig. 5). This suggests a requirement for an injury-induced VCS gene set and a specific functional requirement for *Nkx2-5* in the regeneration of the VCS network after MI at P1.

Strikingly, cell–cell adhesion genes required for fast conduction and ion channels involved in the depolarization phase were downregulated in P7 MI hearts across both the fibroblast-like and cardiomyocyte-like VCS populations but were maintained or upregulated in the infarcted P1 VCS (Fig. 4e,f). HCR analysis validated this differential expression profile such that eGFP^+^ VCS cells were colocalized with *Slc8a1* (solute carrier family 8 (sodium–calcium exchanger), member 1, encoding NCX1) and *Ctnna3* (catenin α3) expression in the infarcted P1 but not P7 VCS (Fig. 4g,h). However, this spatial analysis also revealed upregulation of these same gene sets in *Cx40*-eGFP-negative regions in the infarcted P7 network (Fig. 4h). In this way, we identified genes involved in maintaining a functioning electrical network at P1, including *Slc8a1*, that were not only downregulated in the non-regenerative P7 *Cx40*^+^ network, but also ectopically expressed in discrete areas in the network that lacked *Cx40* expression.

**Extended Data Fig. 6:**
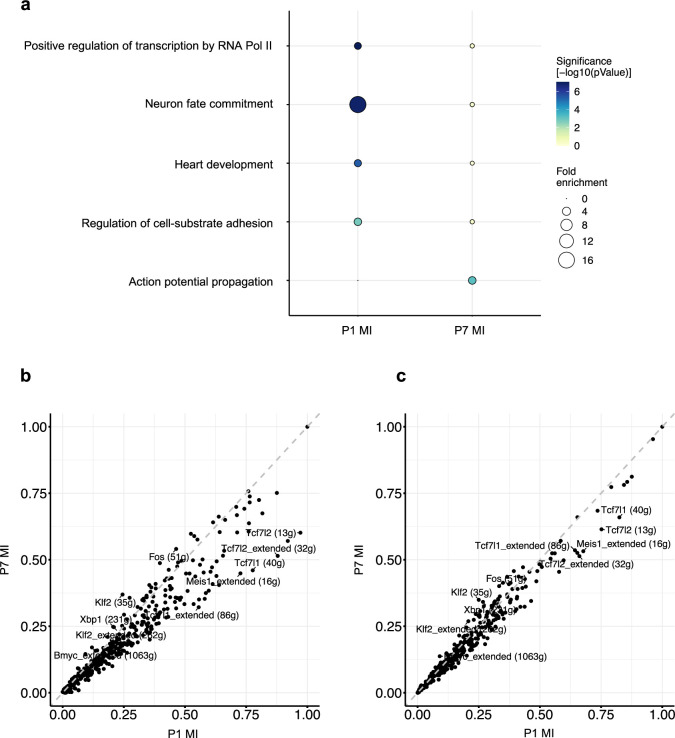
Differential gene regulatory network analysis between the regenerating versus non-regenerating ventricular conduction system. **a**. Dot plot to show the top gene ontology terms associated with the most differentially expressed regulons between the cardiomyocyte-like VCS of P1 MI versus P7 MI hearts (Fisher’s exact test). The same terms were found associated with the most differentially expressed regulons between the fibroblast-like VCS of P1 MI versus P7 MI hearts. **b-c**. The most differentially expressed regulons identified through SCENIC analysis between P1 MI versus P7 MI cells in the cardiomyocyte-like VCS (b) and the fibroblast-like VCS (c) populations.

To identify nodal transcription factors driving the P1 regenerative response, we performed gene regulatory network (GRN) analysis on the full dataset and compared across the VCS of infarcted P1 versus P7 hearts. Using SCENIC^23^, we identified the top regulons of each major cell cluster within the dataset (Supplementary Fig. 10). We found that the most differentially expressed regulons between P1 MI versus P7 MI in the fibroblast-like and cardiomyocyte-like VCS populations were involved in the same pathways: the regenerating P1 VCS upregulated GRNs involved in development, electrical–neuronal fate commitment and modification of cell–substrate adhesion, whereas the non-regenerative P7 VCS upregulated GRNs involved in action potential propagation (Extended Data Fig. 6a). Most notably, the top differentially expressed VCS regulons following MI at P1 versus MI at P7 included *Gata4* (GATA binding protein 4), *Tcf7l1* and *Tcf7l2* (transcription factor-like 1 and 2, T cell specific, HMG box) (Extended Data Fig. 6b). This therefore suggests that Wnt signaling may be involved mechanistically in driving the regenerative response of the P1 conduction system and warrants further study.

Overall, profiling by scRNA-seq identified substantial transcriptional heterogeneity across the postnatal mammalian VCS. Analysis of the regenerating versus non-regenerating VCS identified a shift in cell type composition within the His–Purkinje network and a loss of electrical gene expression in the transition to non-regenerative stages, and implicated NKX2-5 and Wnt signaling in the regenerative VCS response at P1.

### Persistent VCS electrical patho-remodeling

**Fig. 5:**
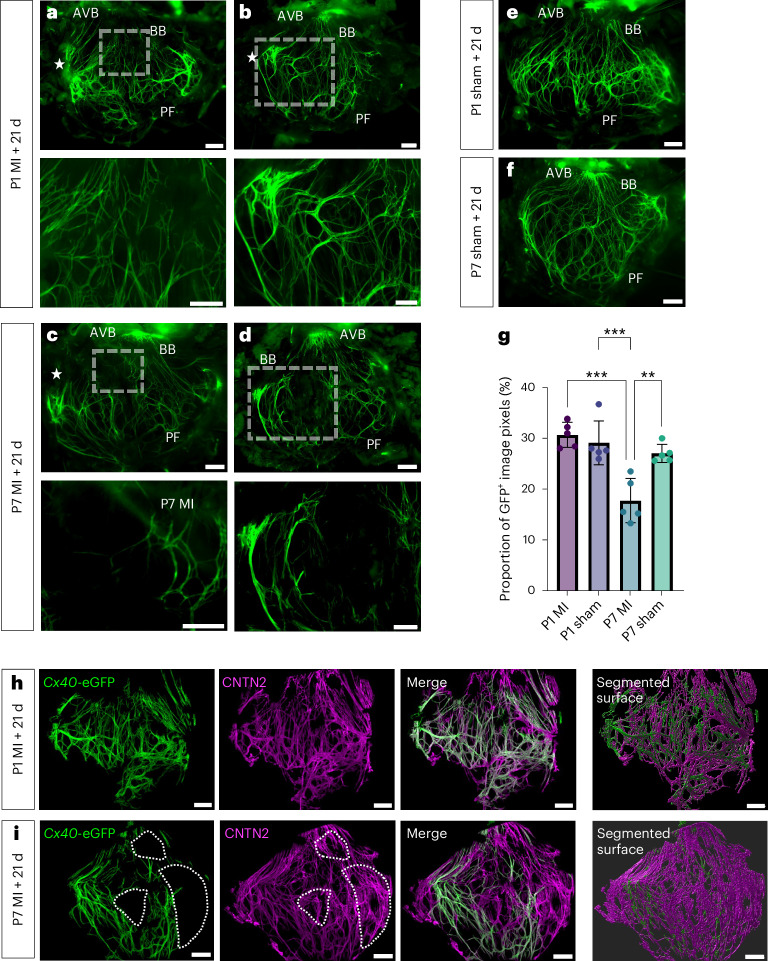
Fast-conducting CX40 is downregulated in patches of the His–Purkinje network following MI in non-regenerative hearts. The LV His–Purkinje network of *Cx40*^eGFP/+^ hearts 21 d following MI (**a**–**d**) or sham surgery (**e**,**f**) at P1 and P7 stages. **a**–**d**, Bottom, magnified inserts from the boxed regions of the corresponding images. While the P1 heart showed enriched *Cx40*-eGFP expression nearest the LAD ligation site but regenerated a full VCS network (**a**,**b**, bottom), the P7 network had patches where *Cx40*-eGFP expression was lost (**c**,**d**, bottom). Stars indicate the level of LAD ligation. Labels indicate the AVB, BBs and Purkinje fibers. Scale bars, 200 μm. **g**, Quantification of the proportion of total pixels in the rectangular region within which the Purkinje fiber network exists, which are *Cx40*-eGFP^+^. The percentage of image pixels positive for GFP is significantly lower in P7 MI hearts than that in all other conditions. Statistics: one-way ANOVA with the Tukey–Kramer test for multiple comparisons (*P < 0.05, **P < 0.01, ***P < 0.001); *P* = 0.0001 (P1 MI versus P7 MI), *P* = 0.0004 (P1 sham versus P7 MI), *P* = 0.0028 (P7 sham versus P7 MI); mean values are depicted; error bars plotted for ±2 s.d.; *n* = 5 independent biological replicates for each condition. **h**,**i**, Immunostaining after fixation for the VCS marker CNTN2 (magenta) and GFP (green) (**h**) revealed that the fibers of the network remained intact but that CX40 was downregulated across patches (dotted regions) remote from the LAD site in the non-regenerative P7 heart after MI (**i**). The experiment was repeated independently three times with similar results. Scale bars, 500 μm, 100 μm for magnified inserts. Source data

**Extended Data Fig. 7:**
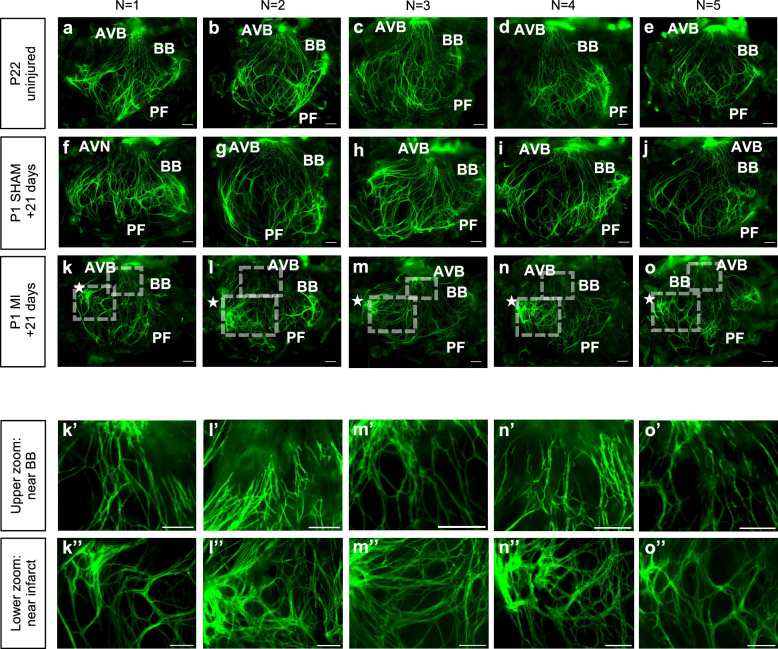
Reconstitution of a full CX40+ conduction network in the regenerative neonatal mouse heart. The dissected left ventricular His/Purkinje network of Cx40eGFP/+ hearts that did not undergo surgery (**a–e**), hearts 21 days following P1 sham surgery (**f–j**), or hearts 21 days following P1 MI surgery (**k-o**). Five representative hearts are shown in each case. Panels show magnified inserts from the boxed regions of the corresponding MI hearts. The P1 heart reconstitutes an apparently normal conduction network with bright CX40+ bundle branches (k’-o’) and full network coverage in the Purkinje fibre area underlying the infarct (k’-o’), but with especially bright CX40+ fibres still apparent nearest the LAD ligation region. Labels indicate atrioventricular bundle (AVB), bundle branches (BB), Purkinje fibres (PF). Scale bars: 500μm, 100μm for zoomed inserts.

**Extended Data Fig. 8:**
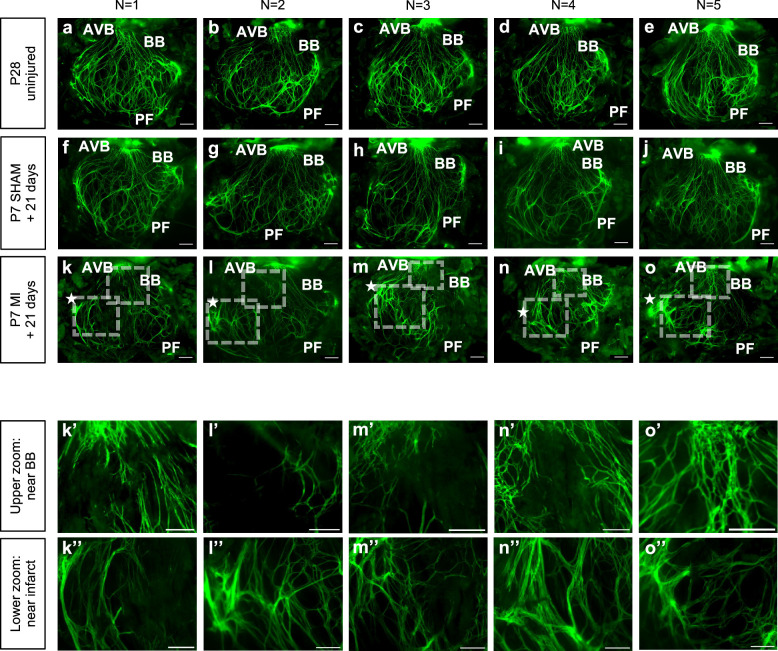
Large gaps in the CX40-eGFP+ bundle branches and Purkinje fibres of the conduction network are sustained in the non-regenerative infarcted mouse heart. The dissected left ventricular His/Purkinje network of Cx40eGFP/+ hearts that did not undergo surgery (**a–e**), hearts 21 days following P7 sham surgery (**f–j**), or hearts 21 days following P7 MI surgery (**k–o**). Five representative hearts are shown in each case. Panels show magnified inserts from the boxed regions of the corresponding MI hearts. The P7 heart fails to reconstitute a normal conduction network: there are large CX40- negative gaps in the bundle branches (k’-o’) and altered network bundling with gaps extending in some cases to the distal Purkinje region, including in the area underlying the infarct (k’-o’). Labels indicate atrioventricular bundle (AVB), bundle branches (BB), Purkinje fibres (PF). Scale bars: 500μm, 100μm for zoomed inserts.

Next, we determined whether the downregulation of regulatory and electrical gene sets identified through scRNA-seq in the P7 VCS after MI results in longer-term remodeling after injury, and whether such changes to electrical identity show regional variation across the VCS network that might predispose to arrhythmia at later stages. To this end, we performed MI or sham surgery on P1 and P7 hearts and allowed 21 d for recovery before harvesting and dissecting open the LVs to expose the His–Purkinje network (Fig. 5 and Extended Data Figs. 7 and 8). In regenerative P1 hearts, there was strong *Cx40*-eGFP expression proximal to the injury site after 21 d (Fig. 5f), but otherwise the VCS appeared morphologically normal, with apparent regeneration of full network architecture and intact bundling of fibers (Fig. 5a–f). However, in non-regenerative P7 hearts, while there was also increased *Cx40*-eGFP expression at the injury site, there were notable gaps in *Cx40*-eGFP expression in the network including in BB regions remote from the infarct area (Fig. 5g–i), which were not present at 3 d after MI (Fig. 2n–p).

To determine whether this absence of signal resulted from loss of VCS cells or from downregulation of CX40 across the network, we performed whole-mount immunostaining for contactin 2 (CNTN2), an alternative VCS cell marker^24^. Positive CNTN2 staining in fibers across the regions lacking *Cx40*-eGFP expression confirmed that VCS cells were not lost, but rather CX40 levels were substantially reduced in discrete areas of the network (Fig. 5m,n). Previous ectopic expression of *Slc8a1* and *Ctnna3* that we identified in CX40-negative regions in the left VCS at 3 d after MI at P7 (Fig. 4h) was also evident as localized puncta of *Slc8a1* and *Ctnna3* signals in the regions negative for *Cx40*-eGFP signal, which were not evident in P1 hearts at 21 d after MI (Supplementary Fig. 11a,b), providing molecular evidence of electrical remodeling in the non-regenerative VCS.

**Extended Data Fig. 9:**
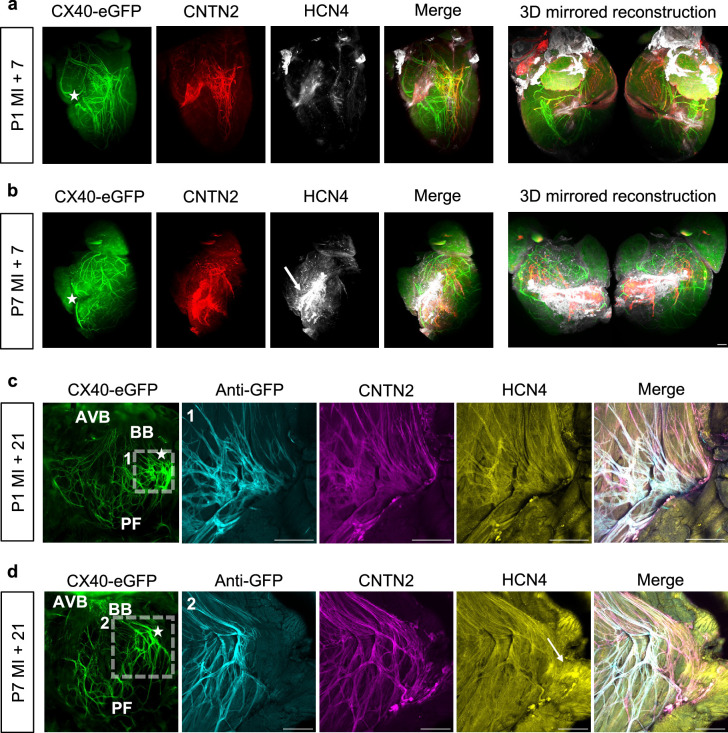
The pace-maker ion channel HCN4 is ectopically expressed in the infarct area after MI and is more dysregulated in non-regenerative hearts. Tissue clearing with immunostaining of wholemount Cx40eGFP/+ hearts 7 days following MI or sham surgery at P1 (**a**) versus P7 (**b**) stages revealed upregulation of HCN4 in the infarct region which was particularly pronounced in the non-regenerative P7 heart. Hearts are shown in 4-chamber view (top) and in side-view (bottom). Stars indicate location of LAD ligation. **c-d**. Immunostaining on dissected left ventricular His/Purkinje network 21 days after MI injury. Antibody staining against GFP (cyan), VCS marker contactin-2 (CNTN2) (magenta) and pace-maker ion channel HCN4 (yellow) revealed upregulation of HCN4 in P7 (d) but not P1 (c) hearts adjacent to the VCS network nearest the site of LAD ligation. Stars indicate location of LAD ligation. Labels indicate atrioventricular bundle (AVB), bundle branches (BB), Purkinje fibres (PF). Scale bars: 500μm.

We further ascertained whether electrical remodeling extended beyond the VCS network to the surrounding myocardium through spatial expression of the pacemaker ion channel hyperpolarization-activated cyclic nucleotide-gated channel 4 (HCN4). HCN4 is normally expressed in the node and at low levels in the VCS but has also been shown to be upregulated in LV working myocytes and implicated as causal for arrhythmias after MI^25,26^. In P1 MI hearts 7 d after injury, HCN4 signal was mainly restricted to the VCS network (Extended Data Fig. 9a), whereas, in P7 MI hearts, there was notable ectopic expression of HCN4 across the infarct area (Extended Data Fig. 9b). Whole-mount immunostaining on the dissected LV His–Purkinje network revealed ectopic HCN4 expression evident in the infarcted myocardium adjacent to the Purkinje network nearest the LAD site in P7 but not P1 MI hearts, 21 d after injury. Additionally, we identified CNTN2^+^HCN4^+^ cells that were *Cx40*-eGFP negative, connected to the *Cx40*-eGFP^+^ network (Extended Data Fig. 9c,d). Our findings reveal ectopic and substantially upregulated HCN4 in infarcted P7 hearts relative to the His–Purkinje network, consistent with an arrhythmogenic phenotype versus maintenance of lower-level, discrete HCN4 expression in the P1 regenerative heart, consistent with preserved normal sinus rhythm.

### The consequence of electrical remodeling for clinical arrhythmogenesis

**Fig. 6:**
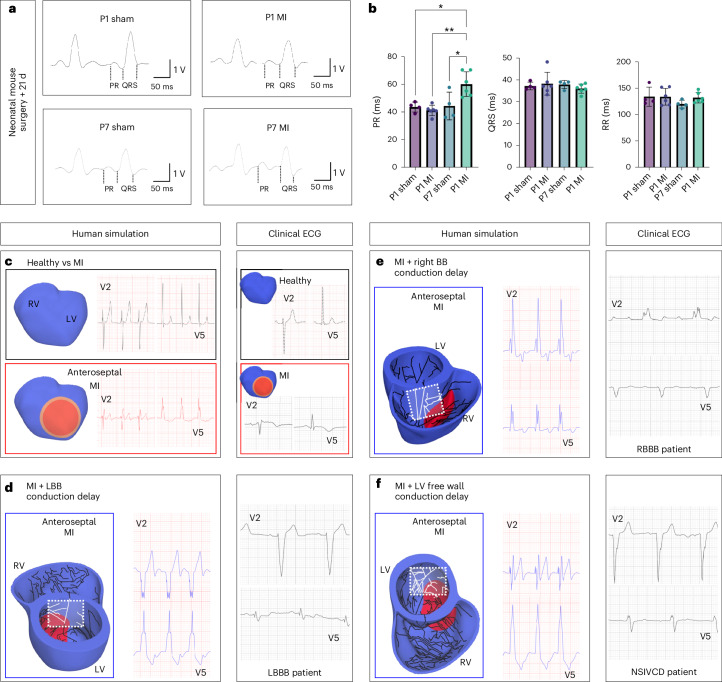
Maintenance of cardiac rhythm in regenerating hearts after MI versus delayed conduction during non-regenerative fibrotic repair. **a**, Representative lead II surface ECGs from mice 21 d after MI or sham surgery at the P1 and P7 stages. ECGs from P7 mice with MI had prolonged PR intervals, whereas ECGs from P1 mice with MI and P1 and P7 sham-operated mice were comparably normal. **b**, Graphs quantifying PR, QRS and RR intervals in mice 21 d after P1 sham, P1 MI, P7 sham and P7 MI surgeries. The PR interval was significantly longer in P7 mice with MI than in all other groups, but no significant differences in QRS or RR intervals were detected, indicating an overall delayed atrioventricular conduction in the non-regenerative infarcted heart. Statistics: one-way ANOVA with the Tukey–Kramer test for multiple comparisons (*P < 0.05, **P < 0.01, ***P < 0.001); *P* = 0.0113 (P1 sham versus P7 MI), *P* = 0.0014 (P1 MI versus P7 MI), *P* = 0.0161 (P7 sham versus P7 MI); mean values are depicted; error bars plot ±2 s.d.; *n* = 6 (P1 MI and P7 MI conditions), *n* = 4 (P1 sham and P7 sham conditions) with independent biological replicates. **c**–**f**, Simulated human ECGs presented alongside analogous clinical patient ECGs (corresponding panels on the right) showing leads V2 and V5 in all cases. **c**, The simulated ECG under healthy conditions closely mirrors the morphology of the ECG from the individual whose anatomy was used to construct the model (black boxes); incorporating an anteroseptal chronic infarct from LAD MI into the model produces an ECG with an inverted T wave, fractured and lengthened QRS and ST deviation (red boxes). ECG alterations are consistent with the clinical ECG in the red box in **c**, right, obtained from a 70-year-old male with hypertension presenting with angina and out-of-hospital VF arrest (treated with cardiopulmonary resuscitation (CPR) and one successful automated external defibrillator (AED) shock) who had occlusion of the proximal LAD and reduced ejection fraction (42%). The patient’s ECG shows T wave inversion and anterior ST (ST segment between ventricular depolarization (QRS complex) and repolarization (T wave)) elevation. **d**, Regionally reduced conduction in a defined patch within the LBB in the simulated anteroseptal MI heart results in an ECG signature typical of LBBB, characterized by prolonged QRS and QRS morphology alterations (leading to QS or RS complexes and notched R waves in lateral leads). These alterations are recognizable in the clinical ECG in **d**, right, obtained from a 69-year-old male with hypertension, type II diabetes mellitus and end-stage renal failure presenting with heart failure symptoms. The patient had large anterior MI with no inducible ischemia on a myocardial perfusion scan. The patient had severely impaired LV systolic function (ejection fraction 30%) and LBBB (QRS duration, 173 ms). **e**, Regionally reduced conduction in a defined patch within the right BB in the simulated anteroseptal MI heart results in an ECG signature typical of RBBB, including QRS prolongation and morphological changes (RSR′ complex in V1 or V2, slurred S wave in lateral leads). This is comparable to the clinical ECG in **e**, right, obtained from a 51-year-old man with type II diabetes mellitus presenting with heart failure symptoms 2 years after coronary artery bypass grafting. The patient had severely impaired LV systolic function on transthoracic echocardiography (previously normal), first-degree heart block (277 ms) and RBBB (QRS duration, 161 ms), despite medical optimization. A myocardial perfusion scan showed a fixed apical perfusion defect, further apical-to-mid-inferior lateral defect, with minimal inducible ischemia. **f**, Regionally reduced conduction in a defined patch of Purkinje fibers in the LV free wall in the simulated anteroseptal MI heart results in an ECG signature typical of NSIVCD (including wide QRS and slurred S wave). Comparable changes are seen in the clinical ECG in **f**, right, obtained from a 63-year-old male with two previous MI events and subsequent coronary artery bypass grafting presenting with worsening heart failure symptoms. Severely impaired function (ejection fraction, 15%) and primary prevention implantable cardioverter defibrillator. Broadening of QRS (160 ms) with NSIVCD. Source data

To determine the functional impact of the observed electrical regeneration versus sustained VCS remodeling, we performed surface electrocardiogram (ECG) recordings 21 d after MI or sham surgery in P1 and P7 mice. We found no significant detectable differences between MI versus sham control groups following P1 surgery. However, P7 MI mice had a significantly prolonged PR interval (time from the onset of the P wave to the start of the QRS complex) as evidence of conduction delay (Fig. 6a,b). Indeed, 50% of P7 animals with MI (*n* = 6) had first-degree atrioventricular block (PR interval of >60 ms^27^), whereas none of the P1 MI or sham-operated animals or P7 sham-operated animals displayed this pathology. These data indicate normal electrical function in the regenerated VCS at P1 versus substantial delays to atrioventricular conduction due to electrical remodeling at P7.

Finally, we addressed the potential consequences of regional downregulation of CX40 and loss of His–Purkinje conduction at non-regenerative stages for arrhythmogenesis in human patients following MI. We conducted simulations with an in silico human ventricular model with Purkinje and healing anteroseptal MI during the first weeks after occlusion. We found that placement of ventricular-like conductivities in localized patches of the His–Purkinje network within the in silico infarcted human heart, mimicking our observations in P7 infarcted mouse hearts, was sufficient to disrupt the normal ventricular activation sequence, ventricular repolarization sequence and ECG signature.

Our simulations reproduced the healthy ECG signature of the patient used to create the ventricular model (Fig. 6c, black box). When anteroseptal infarction was incorporated into the model (Fig. 6c, red box), simulations reproduced ECG abnormalities typical in healing or chronic MI, such as T wave inversion or pathological Q waves. Reduction of conduction in the LBB patch designed to match the experimental findings in the murine LBB resulted in a QRS morphology (ventricular depolarization) typical of a patient with LBBB, characterized by a deep QS complex in leads V1–V3 and ‘M-shaped’, notched or monophasic R waves in the lateral leads (Fig. 6d). To test the impact of equivalent remodeling in the right BB, we next modeled reduced conduction in a patch of right BB network segments and found that this resulted in a QRS morphology comparable to that of patients with right BB block (RBBB) (Fig. 6e). Furthermore, as our experimental data showed reduced CX40 expression not only in the BB but also in more distal regions of the left His–Purkinje network (Fig. 5 and Extended Data Fig. 8), we modeled reduced conduction in an LV free wall His–Purkinje patch (Fig. 6f). Placement of ventricular-like conduction in a local area of free wall network segments resulted in fractures and widened QRS, comparable to those seen in patients with nonspecific intraventricular conduction delay (NSIVCD) (Fig. 6f, right).

**Extended Data Fig. 10:**
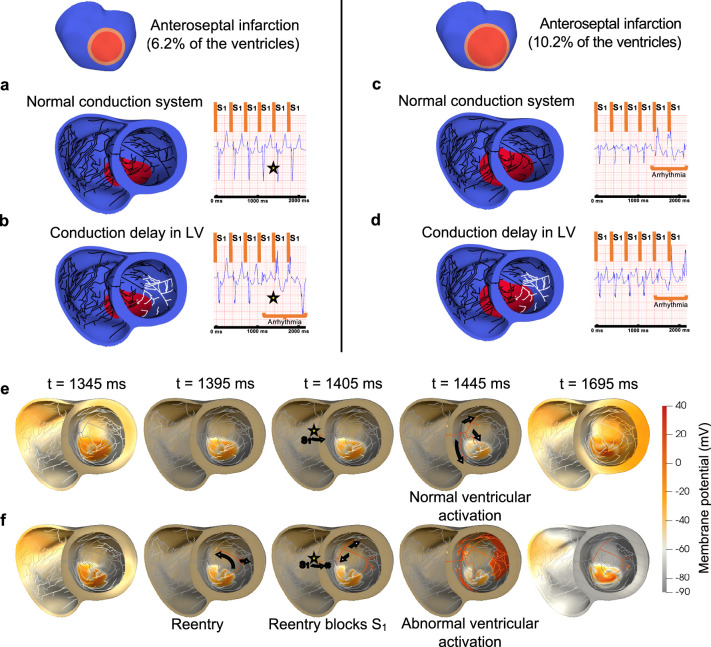
Increased arrhythmic risk caused by VCS conduction delays in MI. **a–d**. Scenarios of human-based computer modelling and simulation of cardiac electrophysiology presenting variability in the size of the MI region (a, b: 6.2% of the ventricles; c, d: 10.2% of the ventricles) and the presence of VCS conduction delay regions (a, c: no conduction delay; b, d: with conduction delay in the LV free wall, coloured in white). For each scenario, the simulated ECG is presented with annotations describing the sinus rhythm stimuli (S1, as orange vertical lines) and the occurrence of arrhythmias. Cases a and b show differences in arrhythmia inducibility based on the existence of VCS conduction delays. **e-f**. are time series of activation maps of cases a and b in the time window 1345–1695 ms, respectively. Repolarisation from the previous sinus rhythm stimulus takes place in both e and f (t = 1345–1395 ms). Case e shows no arrhythmia, as the Purkinje system remained refractory to stimuli from the MI region, hindering reentry. A new stimulus (yellow star in a and e) propagates normally through the VCS (t = 1405–1445 ms) leading to normal ventricular activation (t = 1695 ms). Case f shows a reentrant pattern based on VCS retrograde propagation during ventricular repolarisation (t = 1345–1395 ms). The reentry was caused by the delayed repolarisation in the MI region, eventually entering into the Purkinje fibres retrogradely. The conduction delay in f allows the VCS to recover excitation, thus enabling reentrant patterns. The new sinus rhythm stimulus (yellow star in b and f) collided with the reentrant wavefront in the VCS (red cross, t = 1405 ms), which perturbed the normal activation sequence, prevented uniform depolarisation leading to arrhythmia (t = 1445–1695 ms).

Arrhythmia inducibility was assessed in simulations considering anteroseptal infarction with MI region covering 6.2% (Extended Data Fig. 10a,b) and 10.2% of the ventricular muscle (Extended Data Fig. 10c,d), with normal and slow propagation speed in the free LV VCS, respectively (white portions of the VCS). The ECGs in Extended Data Fig. 10 show the simulated ECG signal in the last 2 s with sinus regular stimulus activity (S1, 1× CL (cycle length) = 375 ms, 5× CL = 350 ms). Conduction delays introduced subtle ECG abnormalities, such as QRS fracturation and bipolar T waves. Extended Data Fig. 10a shows normal activation, whereas the larger MI insult in the patient in Extended Data Fig. 10c led to an arrhythmia. However, when conduction delays were introduced (Extended Data Fig. 10b,d), arrhythmia was induced for both MI sizes. Membrane potential maps in Extended Data Fig. 10a,b during the timespan in which the patient in Extended Data Fig. 10b developed an arrhythmia reveal the arrhythmia mechanism promoted by conduction delays (Extended Data Fig. 10e,f). Simulations were also conducted that revealed delayed VCS conduction arising from reduced conduction in VCS regions, as observed in the P7 infarcted mouse heart, which resulted in ventricular dyssynchrony (Supplementary Fig. 13).

Overall, our in silico results indicate that reduced His–Purkinje conduction following MI, as we identified in the non-regenerative mouse heart, can explain the ECG signature of patients after MI with conduction delays leading to increased arrhythmic risk and higher ventricular dyssynchrony.

## Discussion

Despite the clinical prevalence of arrhythmias following MI, the molecular mechanisms within the VCS His–Purkinje network underlying their incidence are not well understood. Here, we investigated remodeling and repair of the His–Purkinje network after MI in the context of neonatal mouse heart regeneration. We show that the VCS is damaged following MI at regenerative and non-regenerative stages, but, while the P1 heart regenerates its VCS via cellular contributions from existing surviving CX40^+^ cells, the P7 heart showed sustained aberrant changes, most notably including a shift in cell type composition, altered fiber bundling, localized downregulation of CX40 and significant changes in ion channel expression. The cumulative morphological and molecular anomalies corresponded to functional conduction delay as detected by ECG and an increased incidence of dyssynchrony arrhythmias when modeled against the infarcted human heart.

Since the finding that neonatal mammals are capable of full heart regeneration^8^, substantial progress has been made in elucidating the mechanisms by which cardiomyocytes proliferate and new vasculature forms during the regenerative window. However, while there is associated recovery of ventricular function^28^, whether and how this translates to electrical and conductive maintenance or restoration and how the VCS is affected beyond the regenerative window have not previously been established. A potential injury response in Purkinje cells after MI in the P1 mouse heart^29^ associated with improved recovery in a study involving genetic ablation of cells in the atrioventricular node in otherwise healthy animals, suggested the possibility of latent neonatal CCS plasticity^30^. Comparative assessment of neonatal pigs found similar epicardial activation patterns and electrophysiology between P1 MI and healthy control hearts but slower conduction velocity and delayed apex activation in P7 piglets after MI^31^. While this pig study did not interrogate the underlying cellular or molecular mechanisms, or regeneration in the His–Purkinje network, the electrical recovery of full function at P1 versus conduction delay at P7 can be explained by the changes we identify here in His–Purkinje bundle morphology, gene and protein expression and susceptibility to BB block. Consequently, the molecular pathways we have elucidated underpinning VCS regeneration versus pathological remodeling are likely conserved in larger mammals.

Our findings elucidate the core transcriptional networks driving electrical regeneration versus those underpinning pathological modification in the fibrotic context. We identified substantial transcriptional heterogeneity at single-cell resolution across the VCS, including a previously undescribed fibroblast-like population characterized by expression of canonical VCS markers, collagens, cell–cell adhesion and synaptic markers. Importantly, we found a major shift in VCS cell type composition in infarcted P7 hearts, which may account for the reduced ability to reconstitute and maintain a normal His–Purkinje network. Indeed, the reduction in the cardiomyocyte-like VCS population in P7 MI hearts and transcriptional changes within the fibroblast-like VCS population, including reduced expression of cell–cell adhesion markers, may also explain the altered bundle morphology in which the fibrous sheath failed to hold bundles together as was seen in healthy, sham control and regenerative hearts. Moreover, the loss of a minor cycling population in the non-regenerative MI heart, characterized as the cardiomyocyte-like VCS subtype with a G2M/S signature, may further contribute to the lost ability to repair and repopulate the network at P7.

Previous studies focusing on ischemic damage after MI have found that Purkinje cells can survive in the infarct^32,33^ and are thought to be a source of abnormal automaticity in ventricular tachycardias^2,34^. These studies have focused on current flow between surviving Purkinje cells, the infarcted myocardium and the epicardial border zone, as reviewed in ref. ^35^. By contrast, here we provide evidence of spatially dysregulated conduction including downregulation of CX40 in the His–Purkinje network distal from the infarct. This provides a potential molecular mechanism, which is distinct from localized scar-related reentry and remodeling or direct ischemic damage, to explain BB block and increased ventricular dyssynchrony that present following clinical MI. Findings by quantitative PCR and western blot experiments of reduced total CX40 messenger RNA and/or protein levels in Purkinje fibers in the dog^36^ and in LV fibers of a rabbit congestive heart failure model^37^ provide support for large-scale connexin remodeling being conserved in the non-regenerative stressed VCS. It is possible that this phenomenon is mediated by inflammation, given increasing evidence that inflammatory cytokines can downregulate CX40 and CX43 expression, resulting in impaired cell–cell coupling, reduced conduction velocity and enhanced susceptibility to arrhythmia^38^. Moreover, it is known that healthy myocardial regions undergo electrical and structural remodeling in infarcted hearts when adapting to increased workload due to the incidence of impaired contractility within the scar, which may also be a primary mechanism driving VCS remodeling^39^. In this context, it is also interesting that localized loss of CX40 in the VCS corresponded with specific upregulation of NCX1, indicating not just decreased conduction but also altered calcium handling and potentially increased automaticity in these regions of the network.

We further incorporated the identified mechanisms of regionally reduced conduction in the His–Purkinje network into a human modeling and simulation framework. It is striking that this mechanism alone was sufficient to reproduce strong BB block or NSIVCD signatures in the infarcted heart. Our simulations characterized VCS conduction delays as an additional arrhythmogenic mechanism in the context of MI by exacerbating ectopic stimuli and allowing retrograde reentrant pathways in the Purkinje system that severely interfere with sinus rhythm activation. We also found differences between LBBB, RBBB and NSIVCD pathophysiology in that the location of the conduction delay induced variability in myocardial activation and repolarization synchrony. This may explain the observed differences in the proportion of patients with MI and LBBB, RBBB and NSIVCD who go on to develop heart failure, arrhythmogenesis and early mortality^40–42^.

Here we have defined the pathophysiology of the His–Purkinje network in response to injury and implicated the VCS mechanistically as underpinning specific forms of clinical arrhythmogenesis. This contrasts with the current dogma, which almost exclusively implicates ischemic damage, necrosis and scarring as causative for arrhythmias. Collectively, by elucidating the cellular and molecular changes underpinning reconstitution of the infarcted His–Purkinje network in neonatal hearts versus pathological electrical remodeling during fibrotic repair, our study identifies the mechanisms by which normal cardiac conduction is reestablished during heart regeneration. Precise drug targeting of pathologically remodeled VCS cells to restore fast conduction through the network may provide a promising avenue for CCS restoration in patients with MI.

## Methods

### Mouse strains

The *Cx40*^eGFP^ (endogenous GFP knockin line), *Cx40-*Cre^ERT2^ (tamoxifen-inducible Cre line), *Nkx2-5*^Cre/+^ (endogenous Cre knockin line) and *Rosa26*^mTmG^ (Cre reporter line) mouse strains have been described previously^43–46^. CD1 female mice were bred with *Cx40*^eGFP/eGFP^ males for imaging and scRNA-seq neonatal surgery experiments; CD1 female mice were bred with C57BL/6 males for ECG experiments. All animal experiments were carried out in accordance with UK home office project license PDDE89C84 and were compliant with the UK Animals (Scientific Procedures) Act 1986.

### Neonatal myocardial infarction LAD surgery

The neonatal MI procedure was performed as described previously^47^. In brief, mouse litters were separated from their mother and placed in an incubator at 35 °C. General anesthesia was induced (short exposure to 4% isoflurane in oxygen), followed by immersion on ice to induce hypothermal cardiorespiratory arrest. A horizontal incision was then made with surgical scissors at the left mid-thorax. Thoracotomy was achieved using sharp dissecting forceps applied to the third intercostal space near the costochondral junction. The space was widened with blunt forceps, and the heart was extruded from the thoracic cavity by applying gentle pressure to the thorax and the diaphragm while holding the ribs open. An 8-0 Ethilon nylon suture was passed through the heart muscle below the left atrium. Two throws of a surgeon’s knot were tied to ligate the LAD coronary artery in the case of MI surgeries, but no knot was tied for sham procedures. The heart was then gently pressed back into the chest cavity, and the ribs and skin were closed in layers using a 7-0 Prolene suture. The neonates were recovered under an infrared lamp, with administration of oxygen via the nose cone on return of respiration. Warmed pups, once motile and breathing independently, were cleaned and then returned to the dam. Mice were housed and maintained in a controlled environment. All surgical procedures were performed in accordance with the Animals (Scientific Procedures) Act 1986 (Home Office, UK).

### Lineage tracing

*Cx40*-Cre^ERT2/+^ males were mated with *Rosa26*^mTmG/mTmG^ females. Tamoxifen (Sigma-Aldrich, T5648) was diluted to 13.6 mg ml^−1^ in peanut oil, with shaking overnight at 37 °C. Neonates were injected intraperitoneally at P1 with a dose of 0.17 mg per g, followed by neonatal MI surgery at P2 (as described above). Hearts were then collected at day 4 after injury and imaged in whole mount as described below.

### CUBIC tissue clearing with immunostaining

Neonatal hearts were washed with PBS, and the blunt end of the forceps used to apply pressure to the ventricles to pump out residual blood. After shaking in PBS for 30 min, hearts were fixed in 4% PFA at 4 °C overnight. Hearts were washed with 1× PBS at room temperature before incubation in CUBIC-L with shaking at 37 °C. Hearts were incubated in CUBIC-L for at least 3 d, and CUBIC-L was replaced daily. Older postnatal hearts from P7 onward remained in CUBIC-L for a further 8 d, and the CUBIC-L solution was replaced every other day. Hearts older than P14 remained in CUBIC-L for a further 5 d. Hearts were then washed three times with PBS at room temperature for 2 h and transferred to a blocking solution of 0.2% (vol/vol) Triton X-100, 2% (wt/vol) BSA and 10% serum in PBS overnight at 4 °C. Primary antibodies were added for 3 d at 4 °C. Following ten wash steps with PBT (PBS with 0.1% Triton-X), secondary antibodies were added for 2 d at 4 °C. Following seven wash steps with PBS, hearts were moved to CUBIC-R solution. Hearts remained in CUBIC-R for at least 4 d before imaging, shaking at room temperature, and CUBIC-R was replaced every other day. Hearts were imaged in CUBIC-R solution directly. Younger hearts up to P4 were imaged on an Olympus FV1000 inverted confocal in a Lab-Tek eight-well imaging chamber. Older postnatal hearts were imaged in CUBIC-R on the Miltenyi–LaVision BioTec light sheet fluorescence microscope (UltraMicroscope II, Miltenyi Biotec). Hearts were mounted and suspended in CUBIC-R mounting solution (RI 1.520) and attached between two screws to the Miltenyi–LaVision mounting chamber.

### Dissection of the ventricular conduction system and whole-mount imaging

*Cx40*^eGFP/+^ and *Cx40*-Cre^ERT2/+^;*Rosa26*^mTmG/mTmG^ hearts were harvested, and dissecting forceps were used under a fluorescent Zeiss SteREO Discovery.V8 microscope to remove the left atrium; the forceps were inserted directly underneath where the atrium was removed from and used to prize open the ventricular chamber by cutting along the middle of the LV free wall without disturbing the conduction network. Either side of the ventricle wall was folded outward and pinned open at apex and base onto a 4% agarose plate, such that the AVB was exposed centrally at the top of the ventricle, the septal network was centrally located running below the AVB and the free wall distal Purkinje fibers were exposed on left and right sides. The VCS was imaged in this configuration, and samples were then fixed in 4% PFA at 4 °C for 5 h.

Samples were washed at room temperature in PBT (0.3%) and then blocked for 1 h at room temperature in a solution of 0.3% (vol/vol) Triton X-100, 1% (wt/vol) BSA and 10% serum in PBS. Primary antibodies were added in block overnight at 4 °C. Hearts were washed 5+ times with PBT (0.3%) and then 3+ times with PBS. Secondary antibodies were added in PBS overnight at 4 °C, and then samples were further incubated at room temperature for 30 min. After the PBS washes, hearts were immersed in a 1:1 glycerol:PBS solution. Hearts were imaged in the 1:1 glycerol:PBS solution in an ibidi 35-mm glass-bottom imaging chamber. Next, 4% agarose blocks were used as weights to press the dissected hearts flat from above, with the open chamber surface facing downward. Hearts were imaged on a Zeiss LSM 980 inverted confocal microscope.

### Hybridization chain reaction RNA fluorescent in situ hybridization

Whole-mount in situ HCR RNA-FISH was performed using the commercially available methodology from Molecular Instruments. The HCR RNA-FISH protocol for whole-mount mouse embryos (revision 9) was performed in general accordance with the manufacturer’s instructions but with omission of methanol dehydration steps to retain endogenous *Cx40*-eGFP fluorescence. Hearts were imaged in a 1:1 glycerol:PBS solution as detailed above.

### Image analysis

Whole-heart tiled confocal images were stitched in Olympus software or in ZEN Blue software with 10% overlap.

Three-dimensional rendering, background subtraction, segmentation and quantification of whole-mount cleared hearts was performed in Imaris v.10.0. In brief, a region of interest was defined in the *xyz* planes to cover the full ventricular area in which Purkinje fibers existed. Segmentation was performed, thresholding according to local contrast to subtract background signal, so as to create Imaris surface elements covering the full His–Purkinje network. Artery surface elements were subtracted, and then the resulting surface volume was masked to a new channel, keeping original voxel values within the surface volume but assigning all other voxels a zero value. The 3D filament model of the network was then built using the Imaris v.10.0 Filament Tracer. The automated autopath algorithm was used to create a looped filament network based on local intensity contrast. Seed points were defined with a minimum diameter of 9.75 µm. Supervised machine learning training rounds were used to remove extraneous linkages between filaments. The left and right filament models were then duplicated to new filament objects to extract chamber-specific quantification statistics.

All other images were analyzed in Fiji–ImageJ. For calculation of the CX40 network proximal:distal intensity ratio, analysis was performed using 2D SteREO Discovery.V8 images. Each image was cropped to exclude regions beyond the VCS and transformed to position the AVB centrally at the top of the image. Two equally sized rectangular regions of interest were defined to cover the upper and lower halves of the image, respectively. The ROI manager tool was used to calculate the sum of pixel intensity values within the defined regions. The ratio of upper:lower summed values was then calculated for each image. For calculation of the proportion of GFP^+^ pixels in the His–Purkinje network images, each image was cropped to exclude regions beyond the VCS and transformed to position the AVB centrally at the top of the image. Thresholding was performed on each image to separate *Cx40*-eGFP^+^ signal from background fluorescence. The number of GFP-positive and GFP-negative pixels was calculated using the Analyze Histogram tool in Fiji. The percentage of total pixels that were GFP^+^ was then calculated for each image.

### Neonatal heart dissociation for scRNA-seq

Eight P1 MI, six P1 sham, five P7 MI and five P7 sham hearts from *Cx40*^eGFP/+^ litters were dissociated for scRNA-seq. Hearts were harvested, the atria and the top of major arteries were removed with forceps, and the blunt end of the forceps was used to apply pressure to the ventricles to pump out residual blood. Hearts were then immediately transferred to a gentleMACS C Tube (Miltenyi Biotec, 130-093-237) and processed in accordance with the manufacturer’s instructions (Miltenyi gentleMACS Neonatal Heart Dissociation Kit protocol), running the 37C_mr_NHDK_1 program on a gentleMACS Octo Dissociator with Heaters. After RBC lysis, cell pellets were resuspended in 100 µl FcX blocking buffer (1:50 in DMEM, 3% FBS) for 10 min on ice. Next, cells were stained with anti-CD31–BioLegend BV421 antibody (1:50 in 100 µl DMEM, 3% FBS) for 20 min on ice, protected from light.

Samples were then labeled with Cell Multiplexing Oligos from 10x 3′ CellPlex Kit Set A (PN-1000261) in accordance with the manufacturer’s instructions (CG000391 Demonstrated Protocol 3: Cell Multiplexing Oligo Labeling) and individual hearts pooled within each condition. Viability staining was performed immediately before progressing to flow cytometry by adding 1 µl of Zombie NIR dye (1:1,000 in PBS) per tube. Cells were then sorted on an Aria Fusion II cell sorter. Gates were drawn using single and combined controls, and GFP^+^CD31^−^ viable singlets were collected into a 2-ml low-bind Eppendorf tube.

After flow cytometry, cell concentration and viability were calculated using a Countess II cell counter. The supernatant was removed, and cells were resuspended in the appropriate residual volume based on concentration. Samples were then immediately processed according to the 10x Chromium Next GEM Single Cell 3′ v.3.1 (Dual Index) with Feature Barcoding Technology for Cell Multiplexing protocol (CG000388 revision C).

### scRNA-seq data analysis

FASTQ sequencing data were processed using the 10x Cell Ranger multi pipeline (version 7.0.0), aligning reads to the mm10 genome. Only singlets correctly assigned to a single heart in demultiplexing were retained at this stage. Downstream analysis was performed in R (version 4.2.0). Individual heart datasets were merged within conditions, and quality filtering was performed using Seurat (version 4.3.0)^48^. In brief, cells in the top 1% quantile, cells with fewer than 200 detected genes and cells with a contribution of over 40% transcripts from mitochondrial genes were excluded from downstream analysis. The relatively high mitochondrial threshold was chosen due to the properties of conduction cells, as conduction cells are known to have a higher-than-average mitochondrial content^49^. In total, 8,778 P1 MI, 12,431 P1 sham, 7,646 P7 MI and 3,922 P7 sham cells were kept for downstream analysis. Data were normalized by total expression, multiplied by a scale factor of 10,000 and log transformed. The scaled expression matrix was normalized using the Seurat ScaleData function and vars.to.regress set to nUMI and nGenes. Datasets were integrated across conditions in Seurat using the IntegrateData function. Principal-component analysis was applied using genes with the most variable expression across the integrated dataset, followed by non-supervised clustering and data visualization using UMAP. Cell type identities were assigned manually by identifying the most highly conserved genes for each cluster and comparison of these with published datasets^19,20^, known expression profiles (including via the Human Protein Atlas; https://www.proteinatlas.org)^50^ and reported functions^51^. Smooth muscle, immune and epicardial-like VCS clusters were removed before rerunning normalization, principal-component analysis and UMAP analysis. Cell cycle analysis was performed using G2M and S phase genes^52^. Differential abundance testing using *k*-nearest neighbor graphs was performed using the Milo package in R^21^. Differential gene expression analysis was performed using Seurat FindMarkers. GRN analysis was performed using SCENIC^23^.

### Surface electrocardiogram recordings

Litters from C57BL/6 males crossed with CD1 females underwent neonatal MI and sham surgery at P1 or P7, and ECG recordings were collected 21 d after surgery. General anesthesia was induced in an anesthetic chamber (4% isoflurane in oxygen) and maintained by delivering 1–2% isoflurane in oxygen via a facemask while the animal was positioned on a heat mat at 37 °C. Both arms and the left leg of the animal were shaved to remove fur, and the limbs were pinned down using sterile needles. Electrodes were attached to the upper right arm and the lower part of the left leg to achieve a lead II orientation and to the upper left arm and lower left leg for a lead III orientation. Electrodes were connected to a Biopac ECG100C amplifier, and ECG recordings were collected using AcqKnowledge v.4.0 software.

### Statistical analysis

Statistical analyses were performed using GraphPad Prism v.9.0 software. An unpaired, two-tailed *t*-test was used for comparisons between two experimental groups. A one-way ANOVA with multiple comparisons was used for comparing more than two groups with the Tukey–Kramer test. Significant *P* values are reported as ***P < 0.001, **P < 0.01 and *P < 0.05. Individual points are plotted together with mean and error bars indicating ±2 s.d.

### Human-based modeling and simulation framework for human myocardial infarction

A multiscale computational framework for simulating human cardiac electrophysiology from ionic currents to electrocardiographic signals in MI was developed to analyze the effects of reduced conduction after MI in patches of the Purkinje network. The biventricular model of human electrophysiology embedded in a torso was built based on an anatomical model from clinical magnetic resonance imaging of a human individual^53^. The resulting volumetric meshes were discretized in regular cubes of 0.3 mm in each dimension. Myocardial fiber orientation was incorporated using a rule-based method^54^.

The human ventricular cell ToR-ORd^55^ and the human Purkinje cell Trovato models^56^, extensively validated using experimental data, were used to represent membrane kinetics (after 100 regular beats at a cycle length of 500 ms to reach steady state). Electrophysiological heterogeneities in the ventricles were incorporated transmurally, with 70% endocardial and 30% epicardial cells, and in the apicobasal direction (10-ms difference) to obtain realistic activation and depolarization sequences. Ionic remodeling in the infarcted and border zones induced by healing MI was simulated as in ref. ^57^.

To model electrical propagation across the VCS and the working myocardium, we used the monodomain model, which is described by a reaction–diffusion equation given by:

1 $$\beta {C}_{\rm{m}}\frac{\partial V}{\partial t}+\beta {I}_{{\rm{ion}}}\left(V,\,\vec{\eta }\right)=\nabla .\left(\sigma \nabla {V}\,\right)+{I}_{{\rm{stim}}},$$

2 $$\frac{\partial \vec{\eta }}{\partial t}=f\left(V,\,\vec{\eta }\right),$$

where *V* is the transmembrane potential, *I*_ion_ is the total ionic current that may also depend on gating variables $\vec{\eta }$, *β* is the surface:volume ratio, *C*_m_ is the membrane capacitance, *I*_stim_ is the current due to an external stimulus and *σ* is the monodomain conductivity tensor. The monodomain model is further equipped with appropriate initial conditions and no-flux boundary conditions: $\sigma \nabla V.\vec{n}=0$ on $\partial \Omega$, where $\vec{n}$ is the normal vector of the surface $\partial \Omega$.

For electrophysiological simulation, we used MonoAlg3D software^58^, which is a high-performance cardiac solver that uses the graphics processing unit to efficiently compute the monodomain equation. ECG signals were simulated with MonoAlg3D based on the pseudobidomain approach from ref. ^59^.

Baseline conductivity in the longitudinal myocardial fiber direction (*ϭ*_ventricular_) was set to 0.26 S m^−1^ to yield a conduction velocity of 65 cm s^−1^ (ref. ^60^). This produced both a realistic activation sequence and QRS complex in simulations (QRS width of 104 ms in control conditions). In line with the experiments conducted in the present study, specifically data collected from mice 21 d after MI before the scar was fully mature^61^, we modeled MI at the healing stage, namely, during the first weeks after MI onset. Electrophysiological alterations were introduced in the infarct region and the border region, implemented by scaling conductances and time constants of ionic currents in the human cardiomyocyte model based on experimental data and as described in ref. ^53^. A summary of the electrophysiological parameters used to characterize the affected myocardium is provided in Supplementary Table 1. Additionally, tissue conductivities in the infarct and border zones were decreased (19 cm s^−1^ after a ~70% reduction) to match the changes in conduction velocity reported from clinical observations^62,63^. Subsequently, ventricular remodeling in simulations of healing MI led to changes in the activation and repolarization sequences, translated into the simulated ECG as the typical abnormalities seen in patients after MI at that stage, such as fractured QRS complexes and T wave inversion^64,65^.

We computed electrical dyssynchrony based on the simulated ECG signal as per ref. ^66^. To calculate LV intraventricular dyssynchrony, we computed the difference between the times to onset of intrinsicoid deflections (downstroke of the QRS complex) in leads aVL and aVF and divided by the QRS duration. Interventricular dyssynchrony was measured by considering leads V5 and V1 instead (if a QS complex was present in V1, V2 was used instead). A scenario with intraventricular or interventricular dyssynchrony greater than 25% was considered dyssynchronous.

### Patient-specific in silico model of the ventricular conduction system

To model propagation across the Purkinje muscle junction (PMJ), we incorporated two additional currents, ${{I}_{{\rm{PMJ}}}}_{\rm{A}}$ and ${{I}_{{\rm{PMJ}}}}_{\rm{R}}$, to consider anterograde and retrograde PMJ delays, respectively. These two currents were computed only on the Purkinje terminals and tissue cells that were coupled within a PMJ site. For each Purkinje terminal, ${N}_{{\rm{PMJ}}}$ tissue cells were coupled by a fixed resistance ${R}_{{\rm{PMJ}}}$ using the following equations:

3 $${{I}_{{\rm{PMJ}}}}_{\rm{A}}=\,\mathop{\sum }\limits_{i=0}^{{N}_{{\rm{PMJ}}}}\frac{\left({V}^{\,\rm{P}}-{V}_{i}^{\rm{T}}\right)}{{R}_{{\rm{PMJ}}}}$$

4 $${{I}_{{\rm{PMJ}}}}_{\rm{R}}=\,\mathop{\sum }\limits_{i=0}^{{N}_{{\rm{PMJ}}}}\frac{\left({V}_{i}^{\rm{T}}-{V}^{\,\rm{P}}\right)}{{R}_{{\rm{PMJ}}}},$$

where *V*^P^ and *V*^T^ are the transmembrane potential of the Purkinje and tissue cells that are coupled within the PMJ site, respectively, ${R}_{{\rm{PMJ}}}$ is a fixed resistance and ${N}_{{\rm{PMJ}}}$ is the total number of tissue cells that are coupled to the Purkinje terminal cell. ${R}_{{\rm{PMJ}}}$ and ${N}_{{\rm{PMJ}}}$ values were calibrated to reproduce anterograde and retrograde PMJ delays in agreement with experiments^67^. Based on the results of the calibration, we used the following values: ${R}_{{\rm{PMJ}}}=500{\rm{k}}\Omega$ and ${N}_{{\rm{PMJ}}}=60$, which resulted in an anterograde PMJ delay of approximately 2 ms and a retrograde PMJ delay of 1 ms in line with values reported in ref. ^68^. More details regarding PMJ modeling can be found in ref. ^67^.

The anatomy of the VCS was produced as per ref. ^69^ to achieve consistent simulated and clinical QRS complexes, as shown in Fig. 6c (black box). Baseline conductivity in the VCS (*ϭ*_Purkinje_) was set to 2.25 S m^−1^ to obtain a conduction velocity of 200 m s^−1^ (ref. ^67^).

### Incorporation of VCS conduction delays in the human in silico model

The post-MI scenarios presenting reduced VCS conductivity regions were designed to include Purkinje branches in areas matching the CX40 loss identified in the experimental data. The size and location of the VCS sections with reduced conductivity were chosen to replicate the distribution shown in the mouse experiments, affecting both the BBs and the Purkinje system. The VCS delay regions were defined as cuboids with a size of 1 × 3.8 × 3.6 cm to simulate LBBB and RBBB, but, for other locations, the dimensions of the cuboid were adapted to match the extension of the endocardium affected. Next, reduced propagation was imposed on the VCS sections within the cuboid. We explored the variability in the VCS conduction delays by simulating various scenarios featuring changes in size, location (including or excluding the BBs, degree of overlap with the MI region) and number of delay regions (Supplementary Fig. 13). We did not include any specific PMJ remodeling in our simulations because of the lack of human experimental data available to include in the model.

Due to the lack of experimental data in the human VCS after MI, we ran a sensitivity analysis to assess the effects of possible conductivity values in His–Purkinje regions with CX40 loss on the ECG. For this, we simulated anteroseptal MI coexisting with conduction delay in the LBB in a region defined to match the CX40 loss seen in non-regenerative P7 mouse hearts following MI (Fig. 5).

Conduction delays simulating CX40 loss were modeled through the reduction of conductivity: mild (*ϭ*_CX40-loss_ = 1.225 S m^−1^), severe (*ϭ*_CX40-loss_ = 0.26 S m^−1^, same as in ventricular cardiomyocytes in the fiber direction) and extreme (*ϭ*_CX40-loss_ = 0.13 S m^−1^) delays were simulated to assess the ECG signature.

### Stimulation protocol in human ventricular simulations

Sinus rhythm was simulated by delivering an initial stimulus in the His bundle that propagated into the BBs, Purkinje fibers and finally the ventricles. The pacing protocol consisted of progressively faster sinus rhythm stimuli (two beats at CL = 500 ms, two beats at CL = 450 ms, two beats at CL = 425 ms, two beats at CL = 400 ms, one beat at CL = 375 ms and five beats at CL = 350 ms). The ECG was analyzed at CL = 500 ms, whereas arrhythmic mechanisms were studied at the shortest CL, still within the physiological range.

### Clinical data

Written informed consent was obtained for the use of anonymized patient case reports.

### Reporting summary

Further information on research design is available in the Nature Portfolio Reporting Summary linked to this article.

## Supplementary information

Supplementary Information Supplementary Figs. 1–12

Reporting Summary

Supplementary Table 1 Scaling factors of ionic current conductances and time constants to model infarct zone and border zone in the healing stage, as described in ref. ^2^. Supporting experimental data can be found in refs. ^3–6^.

Supplementary Video 1 Three-dimensional rendering, segmentation and modeling of the P2 conduction system, related to Fig. 1. Intact, tissue-cleared imaging of a *Cx40*^eGFP/+^ P2 heart showing the whole organ, the segmented VCS signal and the 3D filament model.

Supplementary Video 2 Three-dimensional rendering, segmentation and modeling of the intact P4 conduction system, related to Fig. 1. Intact, tissue-cleared imaging of a *Cx40*^eGFP/+^ P4 heart showing the whole organ, the segmented VCS signal and the 3D filament model.

Supplementary Video 3 Three-dimensional rendering, segmentation and modeling of the intact P10 conduction system, related to Fig. 1. Intact, tissue-cleared imaging of a *Cx40*^eGFP/+^ P10 heart showing the whole organ, the segmented VCS signal and the 3D filament model.

## Source data

Source Data Figs. 1, 2, 5 and 6 Source Data Extended Data Fig. 3 Statistical source data.

## Data Availability

scRNA-seq datasets generated in this paper are located in the GEO repository under accession number GSE245872.

## Code availability

The code developed in this study is freely available at https://github.com/LLRiebel/MonoAlg3D_C-2023. Anatomical models and simulation files are available at https://zenodo.org/records/14009457 (ref. ^70^).
