# Supplementary material for: Cardiac conduction system regeneration prevents arrhythmias after myocardial infarction
Source: Nat Cardiovasc Res. 2025 Jan 3;4(2):163–79. doi: 10.1038/s44161-024-00586-x (PMC11825367; doi:10.1038/s44161-024-00586-x)
Supplement: Supplementary file 1 — Supplementary Figs. 1–12 [file 44161_2024_586_MOESM1_ESM.pdf]

nature cardiovascular research

Supplementary information https://doi.org/10.1038/s44161-024-00586-x

Cardiac conduction system regeneration prevents arrhythmias after myocardial infarction

In the format provided by the authors and unedited

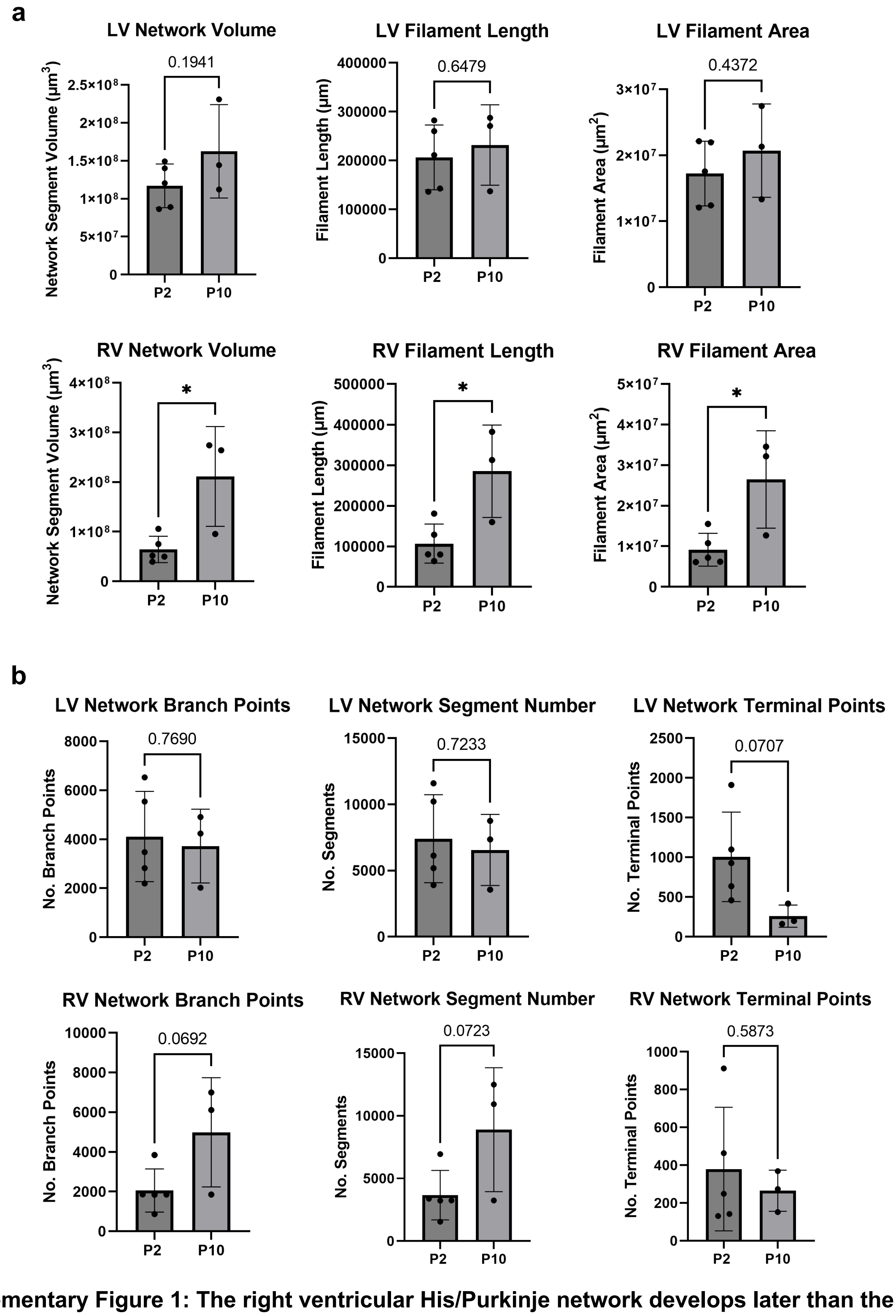

Supplementary Figure 1: The right ventricular His/Purkinje network develops later than the left ventricular network

a. Quantification of the growth of left versus right His/Purkinje networks between post-natal day 2 (P2) and post-natal day 10 (P10). There is a significant increase in right ventricular network volume, total filament length and filament area, but a much more modest increase in the left ventricular network. Statistics: Two-tailed unpaired t test (* $p<0.05$; ** $p<0.01$; *** $p<0.001$); $p=0.0175$ (RV Network Volume), $p=0.0188$ (RV Filament Length), $p=0.0213$ (Filament Area).

b. Quantification of the complexity of left versus right His/Purkinje networks between P2 and P10. There is no discernible difference in extent of network branching or number of segments in the left ventricular network across post-natal development, whereas there is a trend towards increased complexity in the right network over the equivalent time frame. The trend towards reduced number of terminal points between is stronger in the left than the right ventricular network between P2 and P10. Statistics: Two-tailed unpaired t test (* $p<0.05$; ** $p<0.01$; *** $p<0.001$).

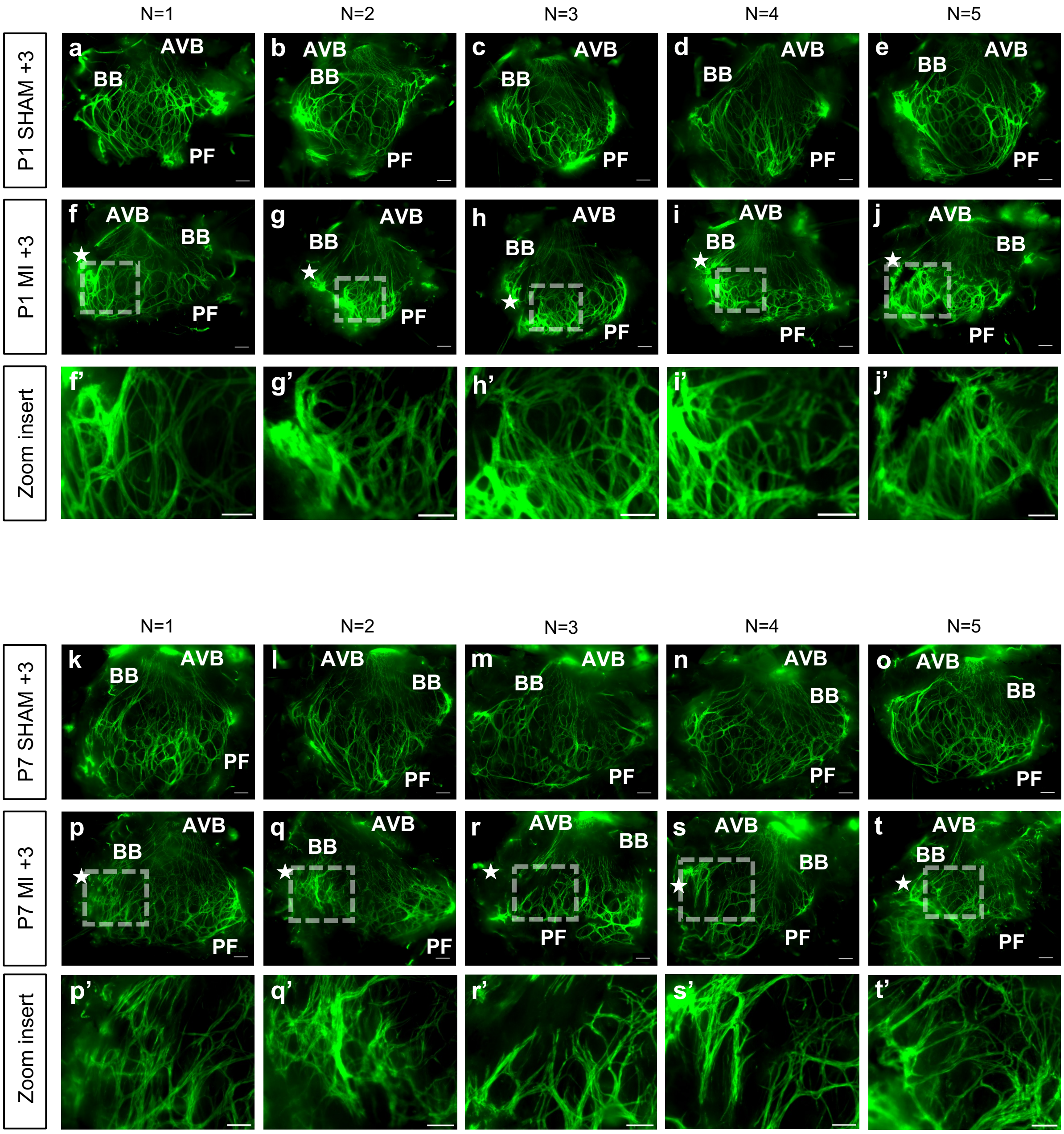

Supplementary Figure 2: Differences in the bundling of conduction fibres after myocardial infarction across five representative hearts

The dissected left ventricular His/Purkinje network of Cx40eGFP/+ hearts 3 days following MI or sham surgery at regenerative P1 (**a-j**) and non-regenerative P7 (**k-t**) stages. Five representative hearts are shown in each case. Panels show magnified inserts from the boxed regions of the corresponding P1 and P7 MI images, highlighting the disruption in fibre bundle morphology after MI. Stars indicate level of the LAD ligation. Labels indicate atrioventricular bundle (AVB), bundle branches (BB), Purkinje fibres (PF). Scale bars: 100μm.

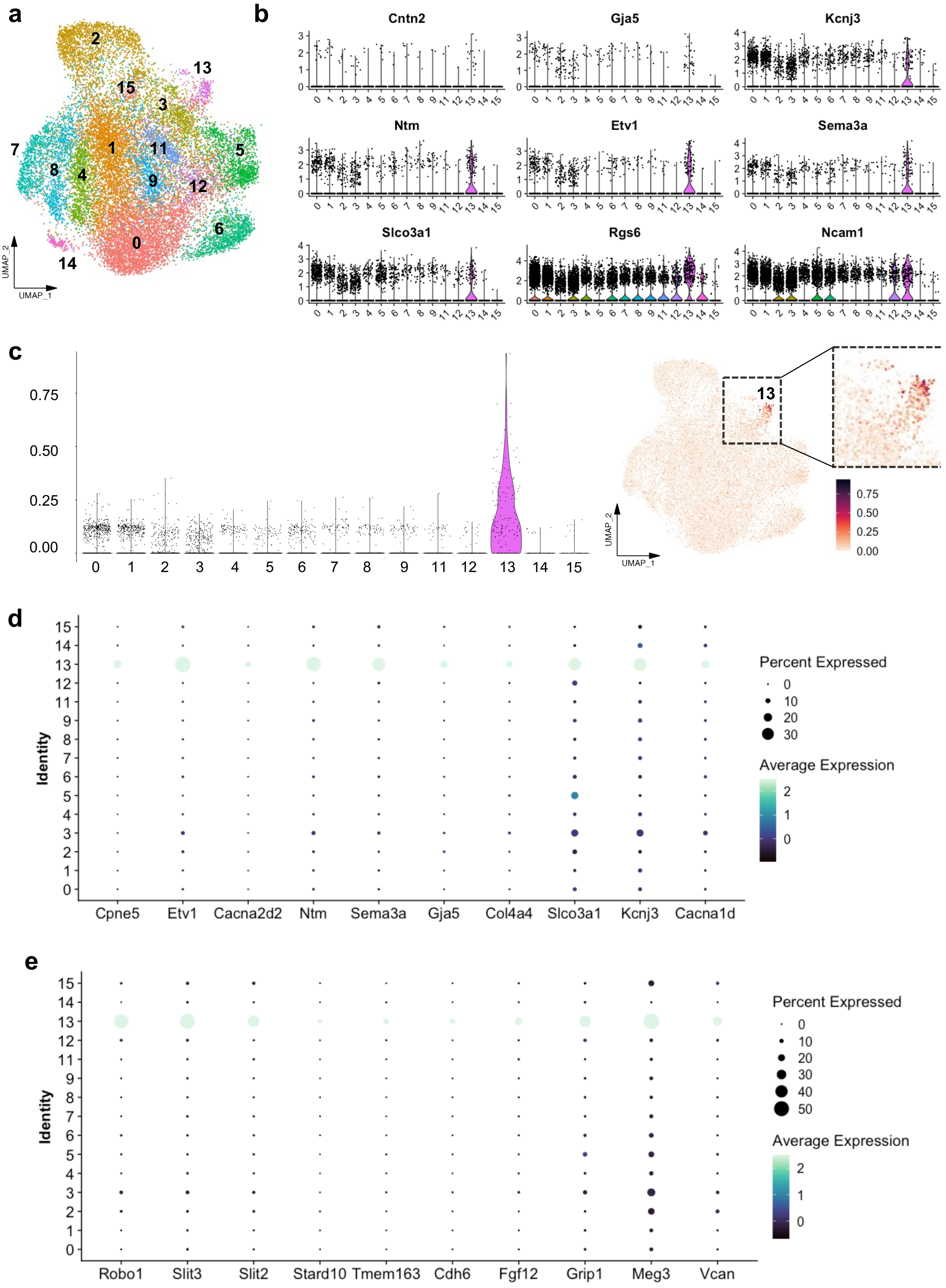

Supplementary Figure 3: Identification and analysis of a Purkinje population in cardiomyocyte-enriched single-nuclear RNA sequencing

a. Uniform Manifold Approximation and Projection (UMAP) visualisation of cardiomyocyte nuclei following re-clustering. **b.** Violin plots show that the ventricular conduction system (VCS) marker expression is upregulated in cluster 13 of the re-clustered dataset. **c**. Violin and UMAP plots of UCell VCS gene set module expression shows upregulation in cluster 13. **d-e.** Dot-plots of gene expression find cluster 13-specific upregulation of known (**d**) and novel (**e**) VCS markers.

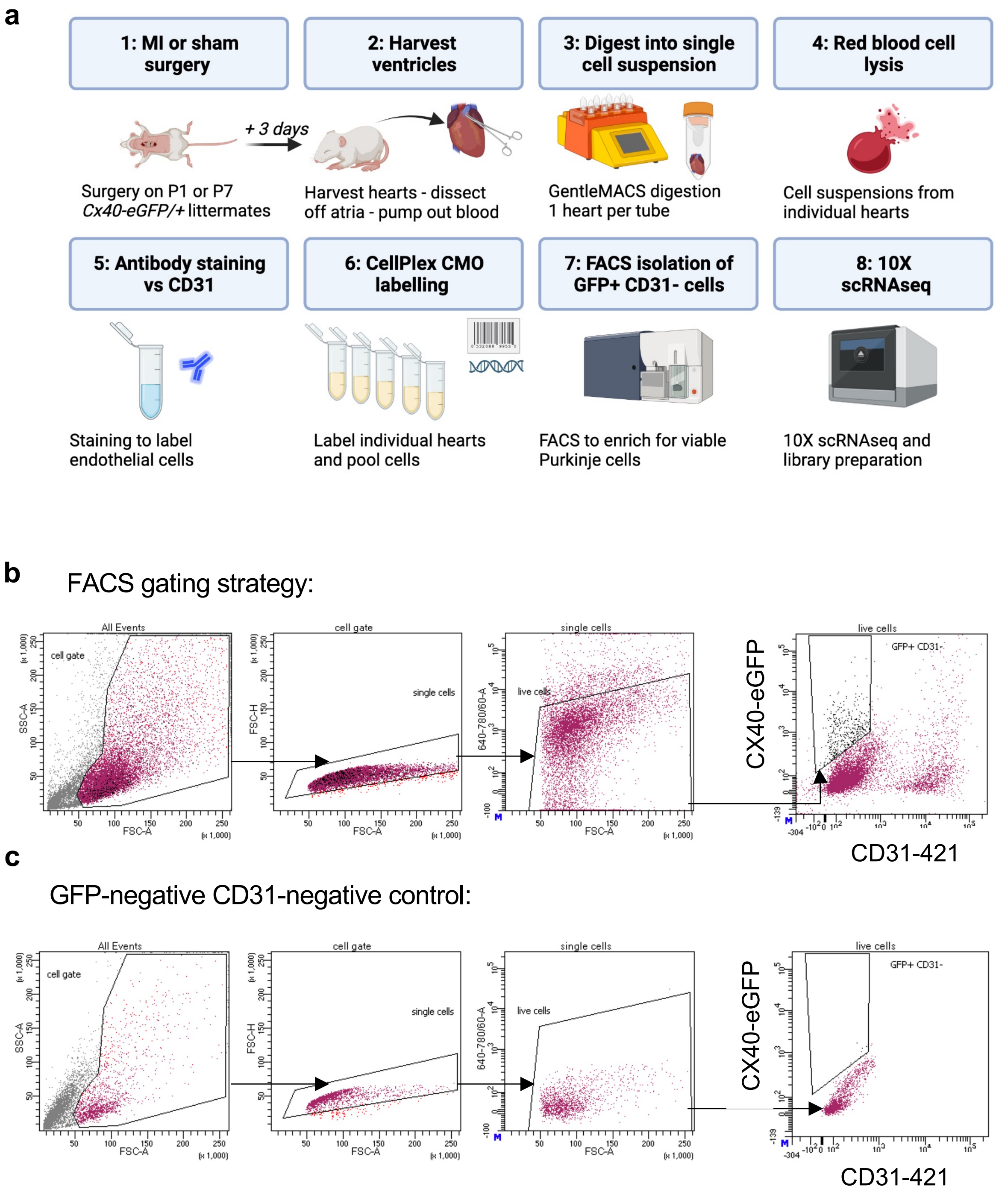

Supplementary Figure 4: A single-cell RNA sequencing strategy to enrich for cells of the ventricular conduction system

a. Schematic to illustrate the stages of sample harvesting, digestion, labelling, ventricular conduction system (VCS) cell enrichment and 10X single-cell RNA sequencing. Created in BioRender. Sayers, J. (2021) https://BioRender.com/r35m095. **b-c.** Fluorescence-activated cell sorting plots showing the gating strategy employed to enrich for viable CX40+ CD31- VCS cells.

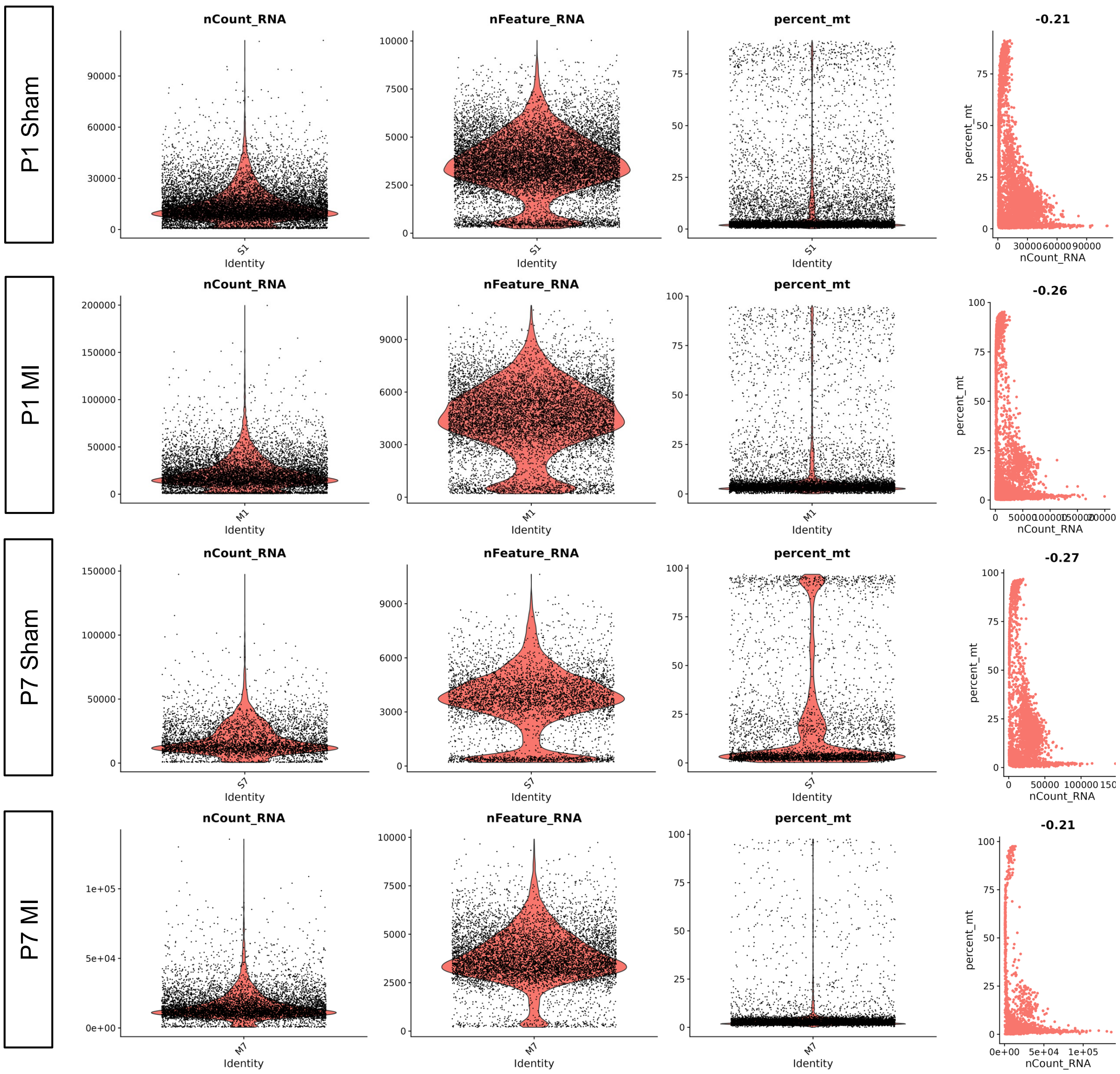

Supplementary Figure 5: Single-cell RNA sequencing quality control
Violin plots showing from left to right: number of unique molecular identifiers; number of genes; percent of mitochondrial reads.

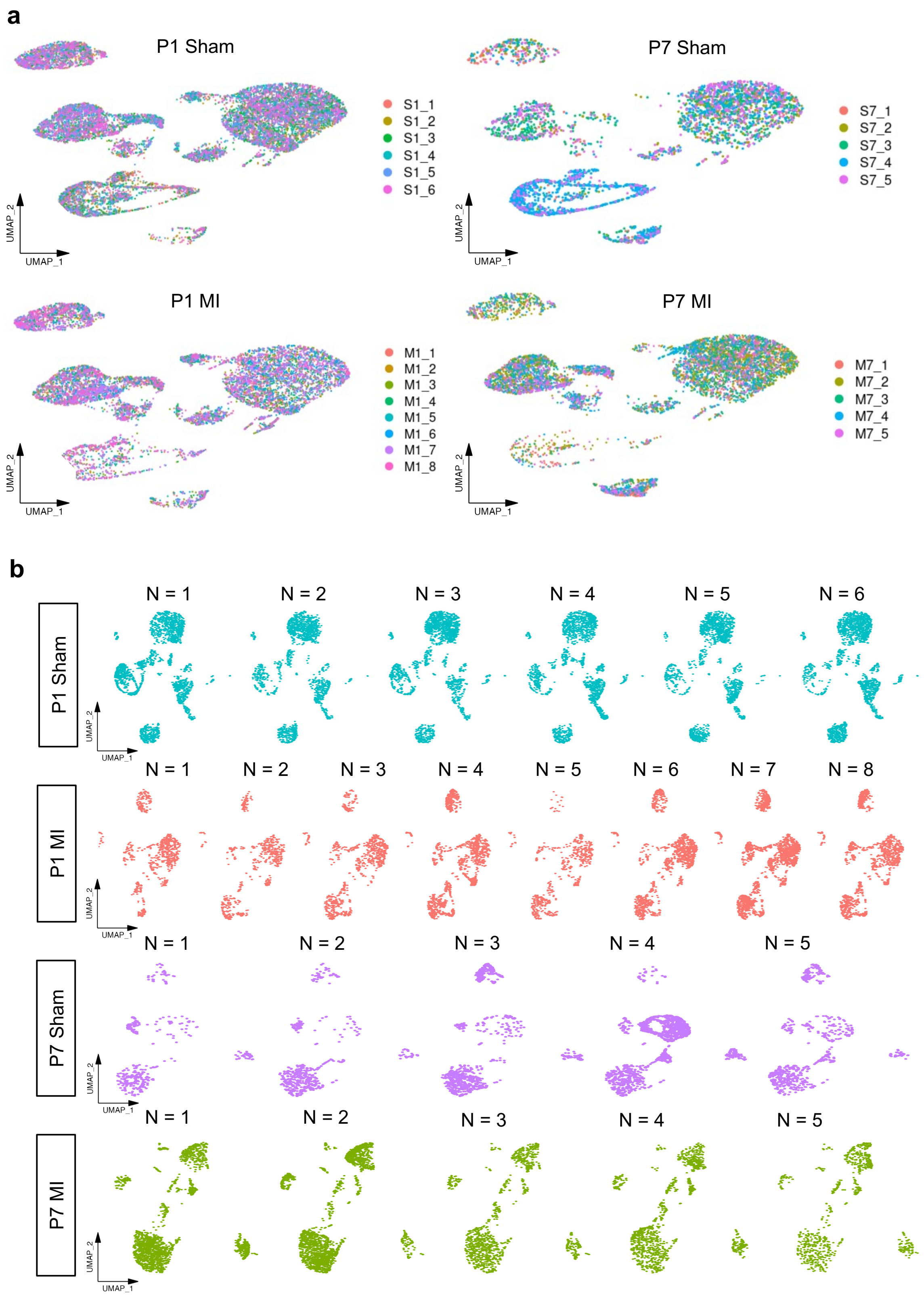

Supplementary Figure 6: Demultiplexing finds no major differences in cell type composition between the Purkinje populations of individual hearts following MI or sham surgeries within the same treatment group

a. Uniform Manifold Approximation and Projection (UMAP) visualisations of P1 sham, P7 sham, P1 MI and P7 MI hearts after integration across conditions. Cells are coloured according to individual heart of origin within each UMAP plot. **b.** Uniform Manifold Approximation and Projection (UMAP) visualisations of P1 sham, P1 MI, P7 sham and P7 MI hearts before integration across conditions, split by individual heart of origin.

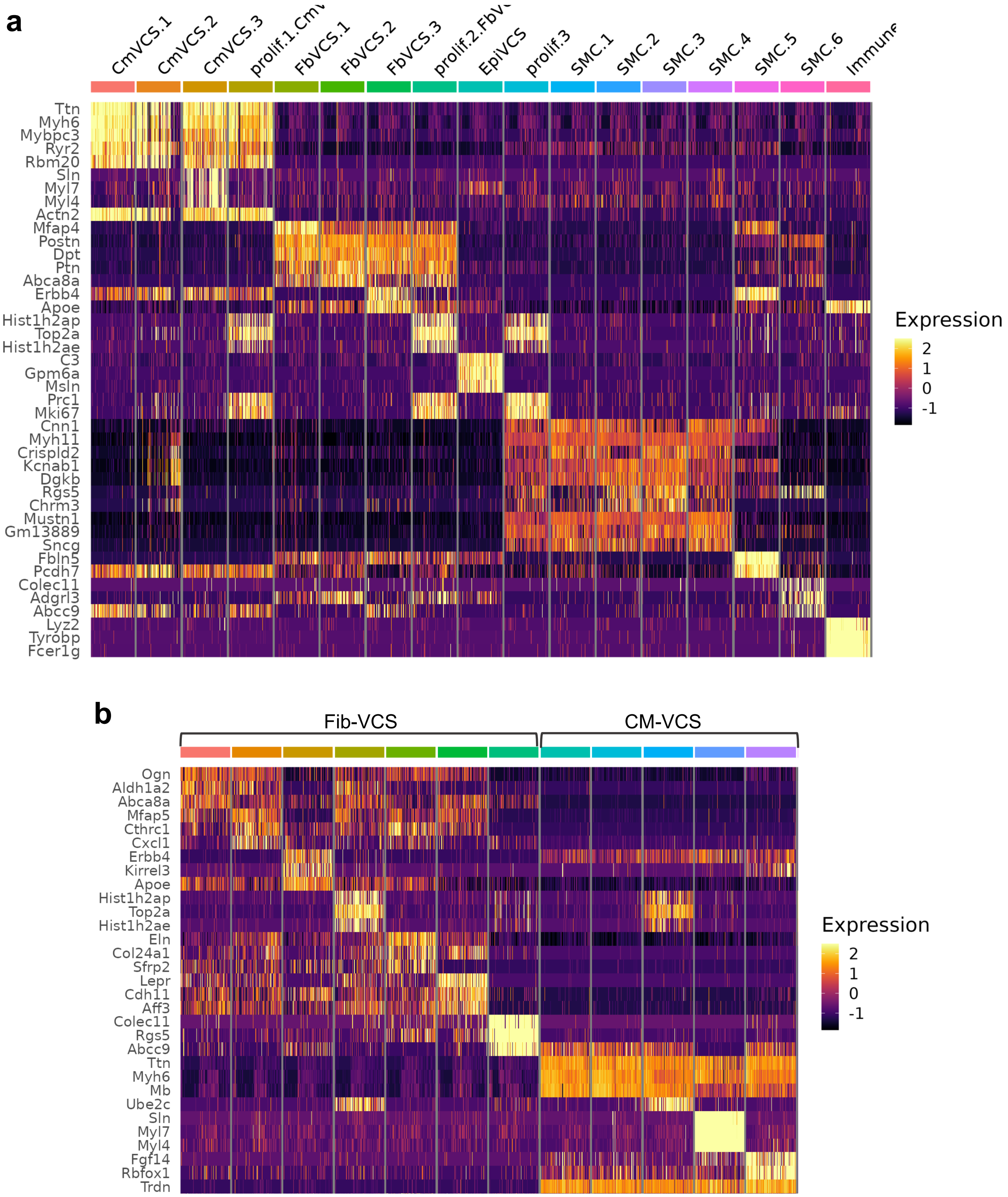

Supplementary Figure 7: Gene expression across the enriched CX40+ CD31- single-cell RNA-sequencing dataset

a. Heatmap showing the top three most highly conserved genes of each cluster in the integrated CX40+ CD31- single-cell RNA-sequencing dataset. **b**. Heatmap showing the top three most highly conserved genes of each cluster in the dataset following re-clustering to remove smooth muscle, epicardial-like and immune cell populations.

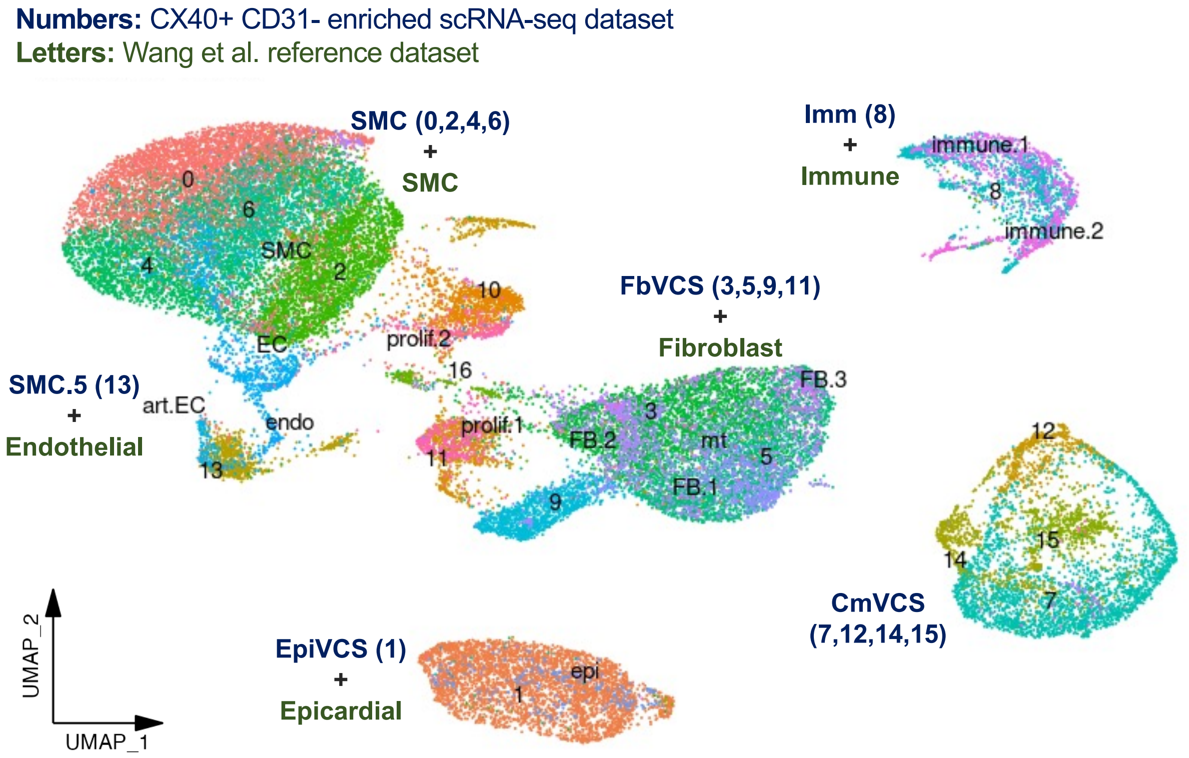

Supplementary Figure 8: Integration of a reference post-natal murine cardiac single-cell RNA-sequencing dataset with the enriched ventricular conduction system dataset

Uniform Manifold Approximation and Projection (UMAP) visualisation following integration of the enriched CX40+ CD31- single-cell RNA-sequencing data with a reference whole heart post-natal murine dataset[1]. Black text labels identify cell types in the reference Wang et al. dataset, black numbers identify clusters of cells from the enriched dataset, green text labels identify wider clusters of the reference Wang et al. dataset, navy text identifies wider clusters from the enriched dataset. The cardiomyocyte-like (CmVCS) populations clustered separately from the reference dataset, the epicardial-like cells (EpiVCS) clustered with the epicardial cells of the reference dataset, and the fibroblast-like (FbVCS) conduction cells clustered with the fibroblast cells of the reference dataset.

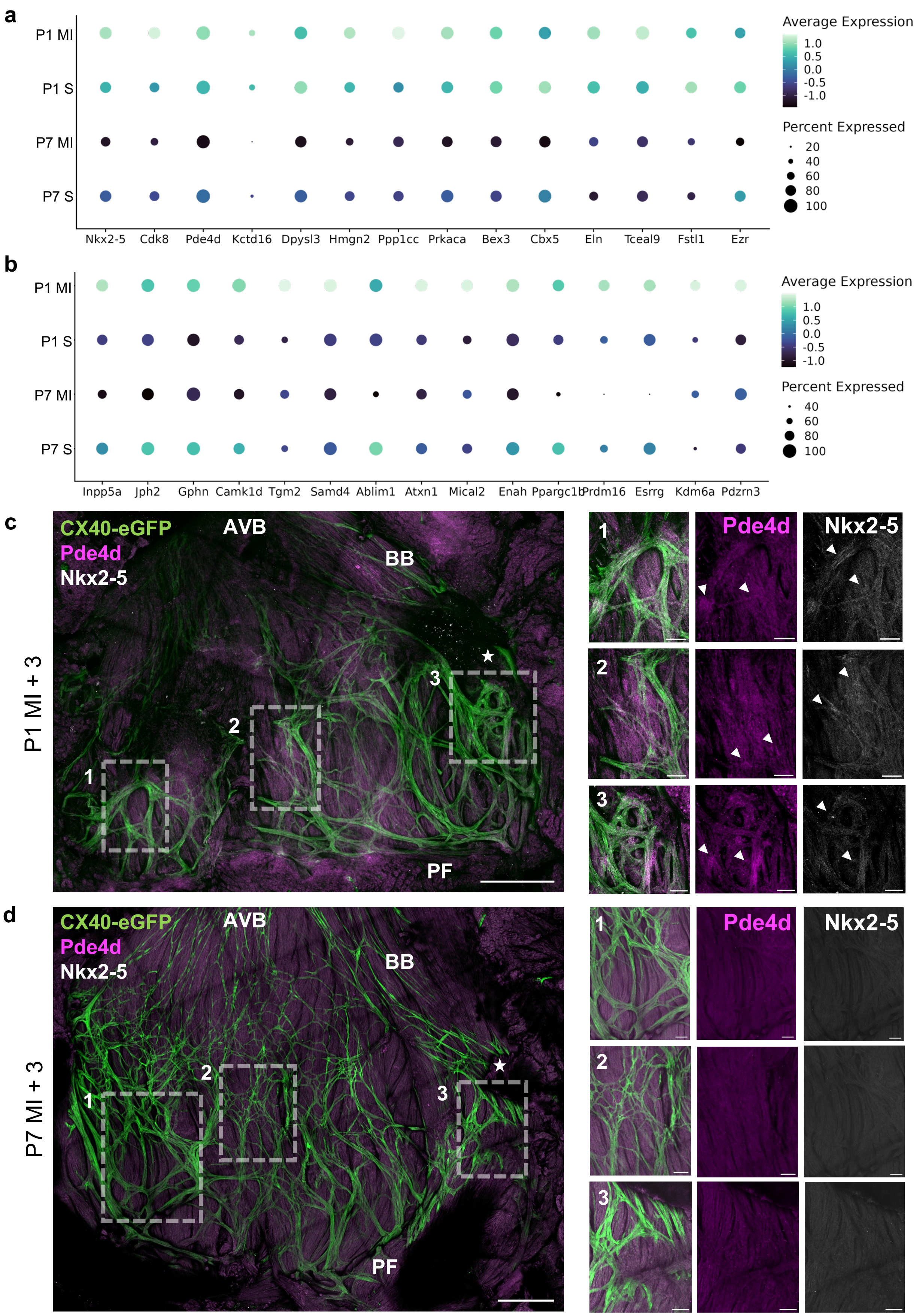

Supplementary Figure 9: Differential expression of gene sets in the regenerative versus non-regenerative His/Purkinje network after myocardial infarction

a. Dot-plot showing average expression of markers with significant differential expression between P1 versus P7 MI hearts within the cluster with most conserved VCS marker expression. **b.** Dot-plot showing

average expression of markers with significant differential expression within the proliferating cluster greatly reduced in P7 MI hearts. In both **a** and **b** genes were analysed that had average log2FC > 0.25, after removing any genes differentially expressed between P1 sham and P7 sham datasets (using log2FC cut-off threshold of 0.1). **c-d.** Hybridisation Chain Reaction (HCR) gene expression analysis of *Pde4d* (magenta) and *Nkx2-5* (grays) on the dissected left ventricular His/Purkinje network of P1 (**c**) and P7 (**d**) hearts, 3-days post-MI. Numbered inserts on the right-hand side show magnified panels from the corresponding boxed regions. Arrows in (**c**) highlight HCR probe expression overlapping with CX40-eGFP-positive conduction fibres in the P1 heart. Experiment was repeated independently 3 times with similar results. Stars indicate location of the LAD ligation. Labels indicate atrioventricular bundle (AVB), bundle branches (BB), Purkinje fibres (PF). Scale bars: 500μm for whole heart images, 100μm for magnified inserts.

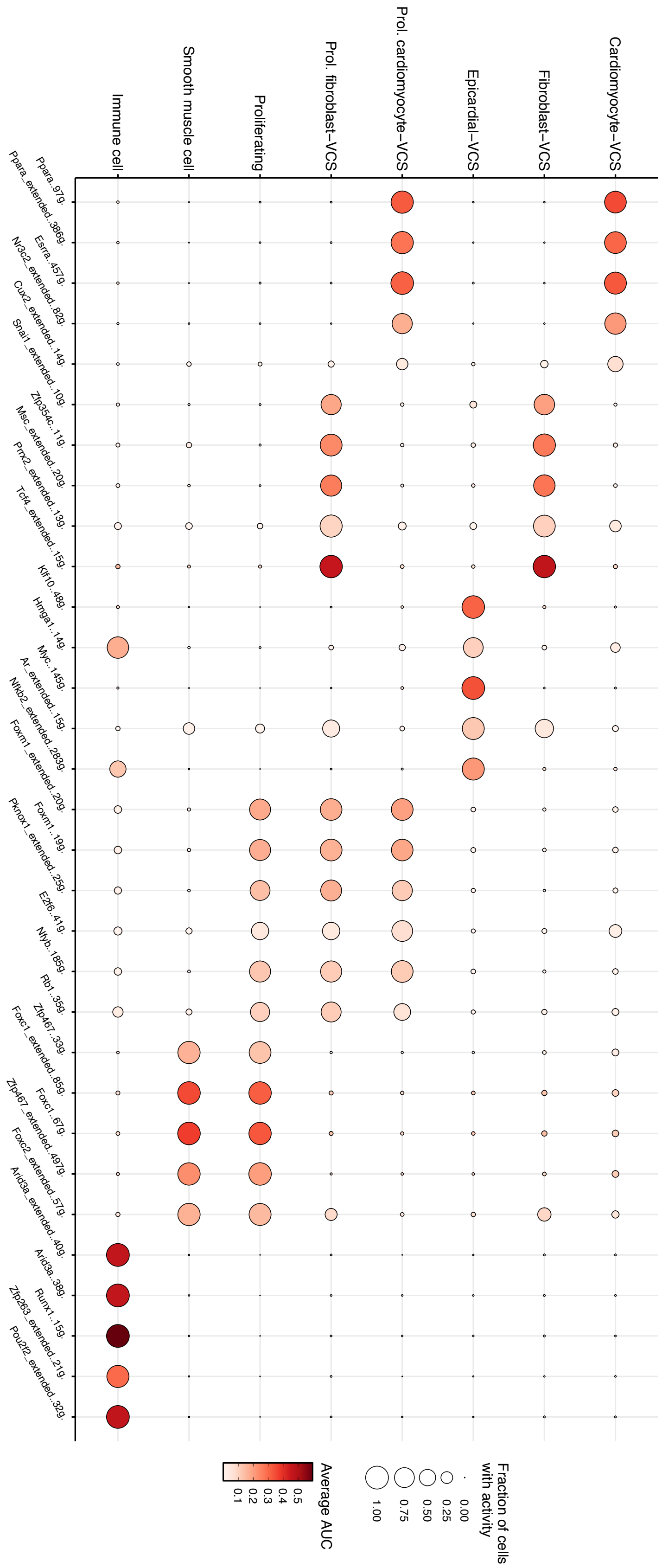

Supplementary Figure 10: The top regulons by major cell cluster in the enriched ventricular conduction system dataset

Dot Plot to show the top 5 regulons across each cluster in the enriched ventricular conduction system single-cell RNA-sequencing dataset, identified through SCENIC gene regulatory network analysis.

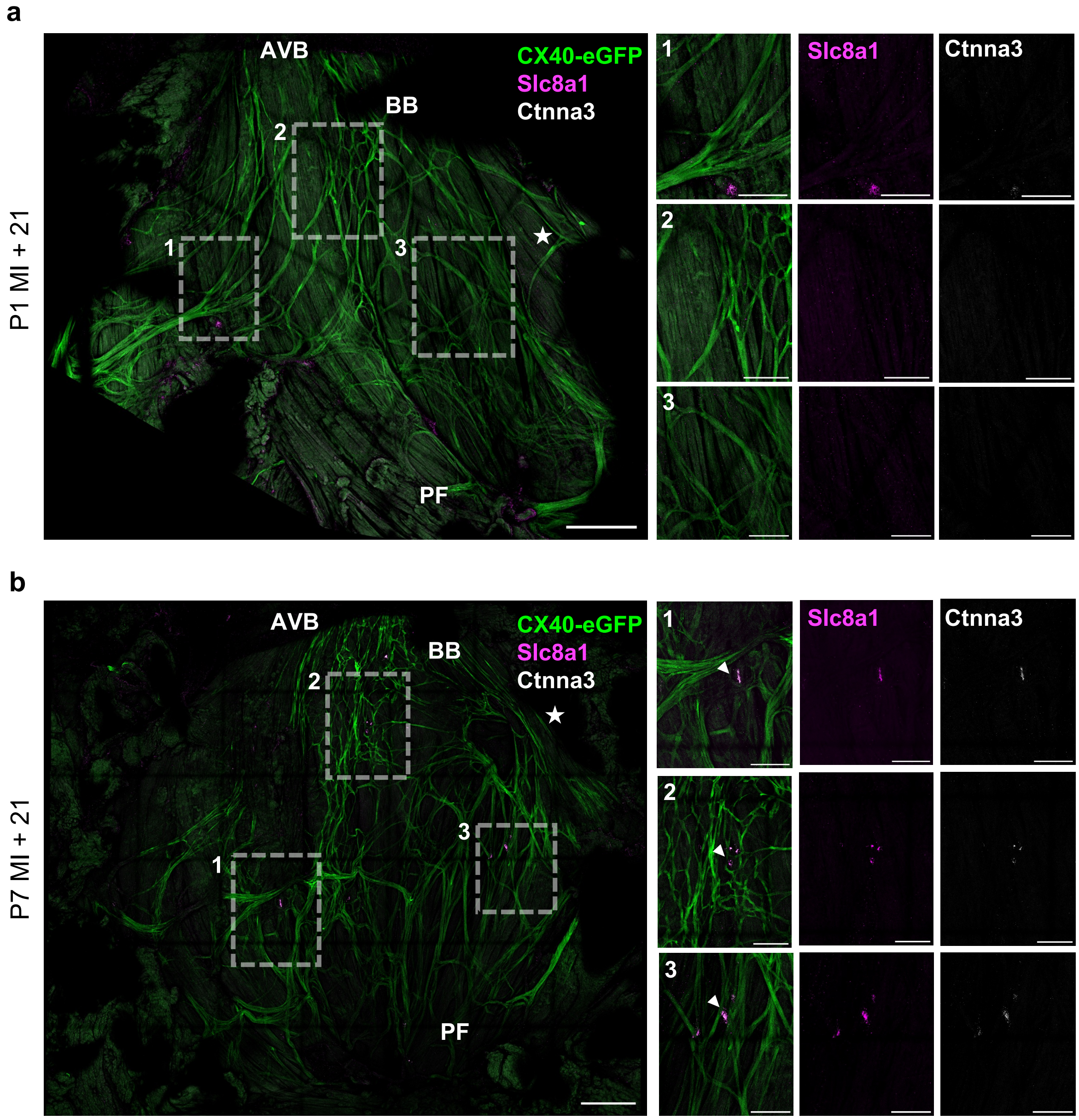

Supplementary Figure 11: Slc8a1 and Ctnna3 are expressed in localized CX40-negative puncta in the non-regenerative infarcted His/Purkinje network

Hybridisation Chain Reaction (HCR) gene expression analysis of *Slc8a1* (magenta) and *Ctnna3* (grays) on the dissected left ventricular His/Purkinje network of P1 (**a**) and P7 (**b**) hearts, 21 days post-MI. Numbered inserts on the right-hand side show magnified panels from the corresponding boxed regions. Experiment was repeated independently 3 times with similar results. Arrows in (**b**) highlight HCR probe expression overlapping with small CX40-eGFP-negative punctate regions in the P7 network. Stars indicate location of the LAD ligation. Labels indicate atrioventricular bundle (AVB), bundle branches (BB), Purkinje fibres (PF). Scale bars: 500μm for whole heart images, 200μm for magnified inserts.

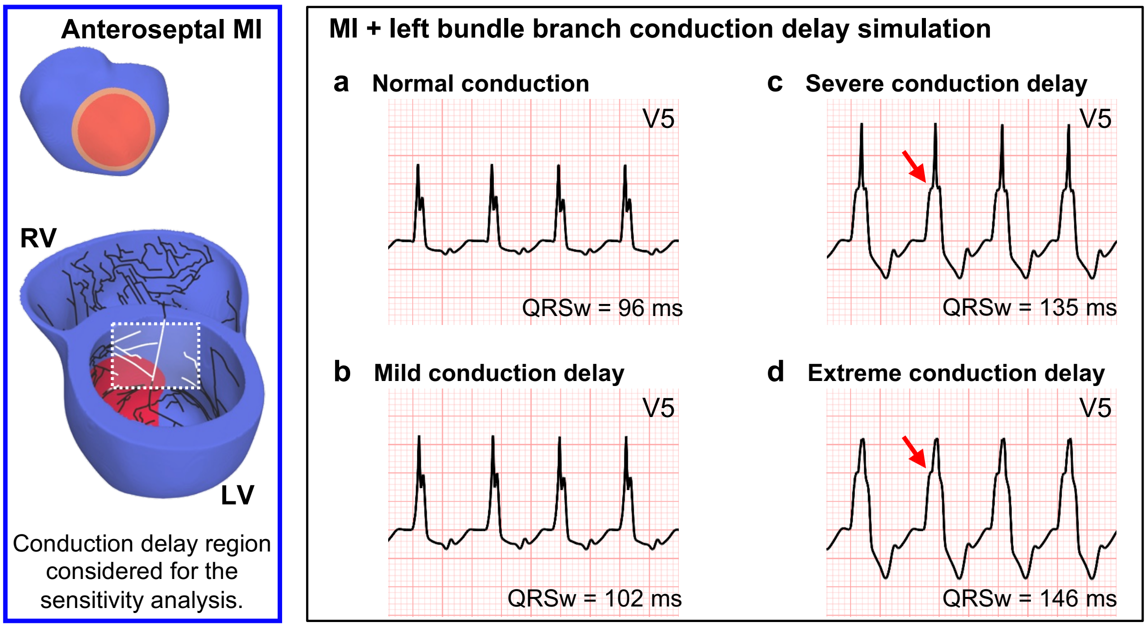

Supplementary Figure 12: Sensitivity analysis for the modelling and simulation of CX40 loss regions in the presence of chronic MI.
Conductivity was reduced at different extents in the VCS section within the dashed blue region to simulate LBB conduction delay. A: healthy conduction (2.25 S/m); B: mild conduction delay (1.255 S/m); C: severe conduction delay (0.26 S/m, the same conduction as the simulated ventricular cardiomyocytes in the fibre direction); and D: severe conduction delay (0.13 S/m). **a** and **b** show simulated ECGs compatible with MI (fractured QRS, T wave inversion). However, **c** and **d** represent a drastically impaired ventricular depolarisation, leading to very prolonged QRS complexes (>120 ms) and notched R waves (red arrows), in addition to MI-derived ECG abnormalities.

	Scenario	ECG signal (V2) (Cycle length = 500 ms)	QRS duration (V2) in ms	ECG dyssynchrony (Katona et al. 2022)		
				intraventricular	interventricular	max dyssynchrony
a	Healthy ventricles<br>No conduction delay<br>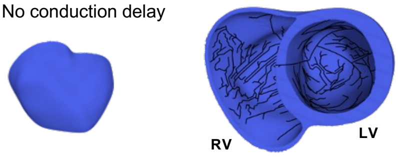	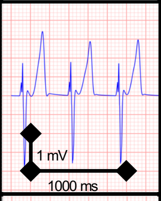	106	12.3%	12.3%	12.3%
b	Anteroseptal infarction<br>No conduction delay<br>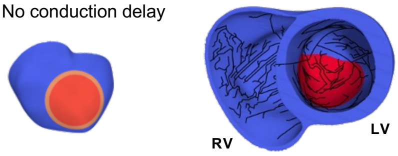	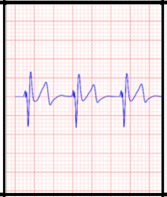	112	7.1%	26.8%	26.8%
c	Anteroseptal infarction<br>Conduction delay overlapping the MI region<br>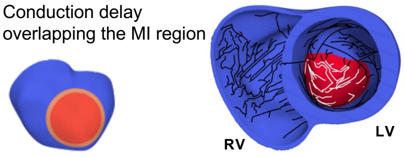	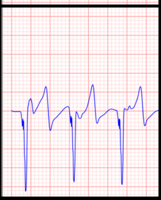	114	13.2%	15.8%	15.8%
d	Anteroseptal infarction<br>Conduction delay in septum including LBB (small region)<br>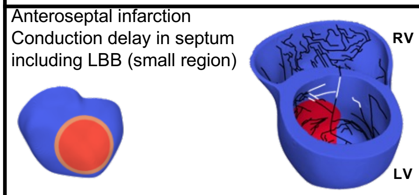	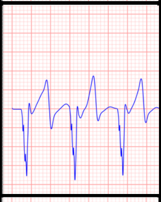	112	25.0%	30.4%	30.4%
e	Anteroseptal infarction<br>Conduction delay in septum including LBB (large region)<br>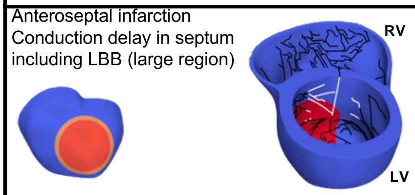	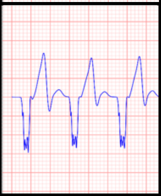	122	24.6%	42.6%	42.6%
f	Anteroseptal infarction<br>Conduction delay in septum excluding LBB<br>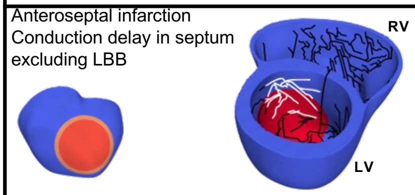	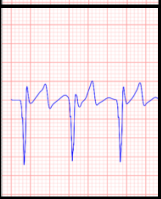	109	14.7%	14.7%	14.7%
g	Anteroseptal infarction<br>Conduction delay in septum including RBB<br>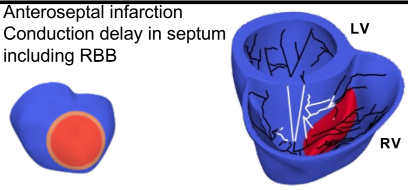	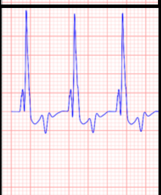	123	8.9%	19.5%	19.5%
h	Anteroseptal infarction<br>Conduction delay in LV free wall<br>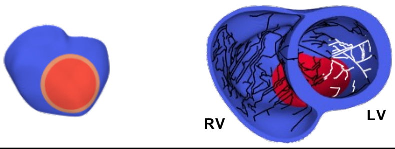	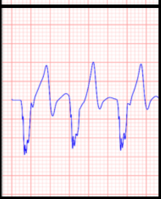	119	10.9%	34.5%	34.5%
i	Anteroseptal infarction<br>2 conduction delay regions: septum (LBB) + LV<br>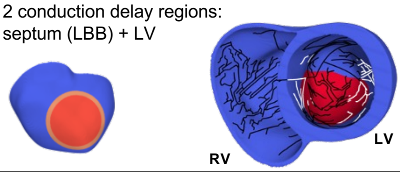	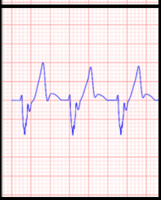	154	20.1%	35.1%	35.1%

Supplementary Figure 13: Set of human-based computer simulations of MI presenting variable VCS conduction delay regions with simulated ECG and annotations on QRS width and ventricular dyssynchrony based on the electrocardiographic signal.
a. Healthy control, no infarction or conduction delay. **b.** anteroseptal MI, no conduction delay. This led to higher electrical dyssynchrony than the healthy control scenario. **c.** anteroseptal MI, conduction delay overlapping the MI region. **d.** anteroseptal infarction, conduction delay including the LBB (1.3 cm affected). **e.** anteroseptal infarction, conduction delay including the LBB (3.8 cm affected). **f.** anteroseptal infarction, conduction delay in the anteroseptal region excluding the LBB. **g.** anteroseptal infarction and conduction delay in the RBB. **h:** anteroseptal infarction and conduction delay in the LV free wall. **i:** anteroseptal infarction and two conduction delays (LBB and LV free wall). Electrical dyssynchrony is detected when either interventricular or intraventricular dyssynchrony is higher than 25% (pink-shaded).
Conduction delays in the VCS led to increased dyssynchrony in most cases, but only LBB or RBB severely affected by the conduction delays lead to QRS width longer than 120 ms, as in patients with conduction blocks (**d, g** and **i**). Delay in LBB was the main factor determining dyssynchrony (**d**: 30.4%; **e**: 42.6%; **i**: 35.1%) since other scenarios with delay regions of similar size and location but excluding the LBBB led to normal dyssynchrony values (**c**: 15.8%; **f:** 14.7%). Surprisingly, conduction delays overlapping with the MI region reduced electrical dyssynchrony (**c**: 15.8%; **f**: 14.7%) imposed by anteroseptal MI (**b**: 26.8%). This is explained by the fact that VCS conduction delays overlapping the MI region impaired depolarisation of the affected area, albeit promoting repolarisation heterogeneities and thus an increased risk of reentry.
The scenario simulating RBBB showed subclinical values of electrical dyssynchrony (**g**: 17.1%), whereas the QRS morphology (RSR’ pattern) and width (123 ms) were representative of RBB patients. Simulation of NSIVCD showed QRS prolongation (119 ms), fractured QRS and high electrical dyssynchrony (**h**: 34.5%). Ventricular dyssynchrony was also determined by the size of the conduction delay region in the VCS: larger affected regions lead to larger dyssynchrony (**d**: 30.4%; **e**: 42.6%) and QRS width (112 ms vs 122 ms).

1 Wang, Z. *et al.* Cell-type-specific gene regulatory networks underlying murine neonatal heart regeneration at single-cell resolution. *Cell reports* **33**, 108472 (2020).
