# Supplementary material for: Cardiac conduction system regeneration prevents arrhythmias after myocardial infarction
Source: Nat Cardiovasc Res. 2025 Jan 3;4(2):163–79. doi: 10.1038/s44161-024-00586-x (PMC11825367; doi:10.1038/s44161-024-00586-x)
Supplement: Supplementary file 2 — Reporting Summary [file 44161_2024_586_MOESM2_ESM.pdf]

nature portfolio

Corresponding author(s): Paul Riley
Last updated by author(s): Nov 3, 2024

Reporting Summary

Nature Portfolio wishes to improve the reproducibility of the work that we publish. This form provides structure for consistency and transparency in reporting. For further information on Nature Portfolio policies, see our Editorial Policies and the Editorial Policy Checklist.

Statistics

For all statistical analyses, confirm that the following items are present in the figure legend, table legend, main text, or Methods section.

n/a	Confirmed	
☐	☒	The exact sample size (*n*) for each experimental group/condition, given as a discrete number and unit of measurement
☐	☒	A statement on whether measurements were taken from distinct samples or whether the same sample was measured repeatedly
☐	☒	The statistical test(s) used AND whether they are one- or two-sided<br>*Only common tests should be described solely by name; describe more complex techniques in the Methods section.*
☐	☒	A description of all covariates tested
☐	☒	A description of any assumptions or corrections, such as tests of normality and adjustment for multiple comparisons
☐	☒	A full description of the statistical parameters including central tendency (e.g. means) or other basic estimates (e.g. regression coefficient) AND variation (e.g. standard deviation) or associated estimates of uncertainty (e.g. confidence intervals)
☐	☒	For null hypothesis testing, the test statistic (e.g. *F*, *t*, *r*) with confidence intervals, effect sizes, degrees of freedom and *P* value noted<br>*Give P values as exact values whenever suitable.*
☒	☐	For Bayesian analysis, information on the choice of priors and Markov chain Monte Carlo settings
☒	☐	For hierarchical and complex designs, identification of the appropriate level for tests and full reporting of outcomes
☒	☐	Estimates of effect sizes (e.g. Cohen's *d*, Pearson's *r*), indicating how they were calculated

Our web collection on statistics for biologists contains articles on many of the points above.

Software and code

Policy information about availability of computer code

Data collection	Please see extensive details in the methods section of the manuscript. In brief, microscopy data was collected on the following: Zeiss SteReo Discovery.V8, Olympus FV1000 inverted confocal, Miltenyi-LaVision Bio Tec light sheet fluorescent microscope. ScRNA-seq data was collected using: Aria Fusion II cell sorter; 10X Chromium Controller; Illumina NovaSeq.
Data analysis	Please see extensive details in the methods section of the manuscript. In brief, image analysis was performed using: Imaris 10.0; Olumpus software; ZEN blue; Fiji/ImageJ. ScRNA-seq analysis was performed using: 10X CellRanger multi (version 7.0.0), R (version 4.2.0), Seurat (version 4.3.0), Milo package in R, SCENIC package in R.Statistical analysis was performed using: GraphPad Prism 9.0 software. Code from this study has been deposited in GitHub: https://github.com/LLRiebel/MonoAlg3D_C-2023. Anatomical model and simulation files have been deposited at Zenodo: https://zenodo.org/records/14009457.

For manuscripts utilizing custom algorithms or software that are central to the research but not yet described in published literature, software must be made available to editors and reviewers. We strongly encourage code deposition in a community repository (e.g. GitHub). See the Nature Portfolio guidelines for submitting code & software for further information.

Data

Policy information about availability of data

All manuscripts must include a data availability statement. This statement should provide the following information, where applicable:

- Accession codes, unique identifiers, or web links for publicly available datasets
- A description of any restrictions on data availability
- For clinical datasets or third party data, please ensure that the statement adheres to our policy

The single-cell RNA-seq data were deposited in the Gene Expression Omnibus under accession no. GSE245872. Data generated or analysed during this study are freely available. Human protein atlas which was used is freely available at: proteinatlas.org.

Research involving human participants, their data, or biological material

Policy information about studies with human participants or human data. See also policy information about sex, gender (identity/presentation), and sexual orientation and race, ethnicity and racism.

Reporting on sex and gender	*Use the terms sex (biological attribute) and gender (shaped by social and cultural circumstances) carefully in order to avoid confusing both terms. Indicate if findings apply to only one sex or gender; describe whether sex and gender were considered in study design; whether sex and/or gender was determined based on self-reporting or assigned and methods used. Provide in the source data disaggregated sex and gender data, where this information has been collected, and if consent has been obtained for sharing of individual-level data; provide overall numbers in this Reporting Summary. Please state if this information has not been collected. Report sex- and gender-based analyses where performed, justify reasons for lack of sex- and gender-based analysis.*
Reporting on race, ethnicity, or other socially relevant groupings	*Please specify the socially constructed or socially relevant categorization variable(s) used in your manuscript and explain why they were used. Please note that such variables should not be used as proxies for other socially constructed/relevant variables (for example, race or ethnicity should not be used as a proxy for socioeconomic status). Provide clear definitions of the relevant terms used, how they were provided (by the participants/respondents, the researchers, or third parties), and the method(s) used to classify people into the different categories (e.g. self-report, census or administrative data, social media data, etc.) Please provide details about how you controlled for confounding variables in your analyses.*
Population characteristics	*Describe the covariate-relevant population characteristics of the human research participants (e.g. age, genotypic information, past and current diagnosis and treatment categories). If you filled out the behavioural & social sciences study design questions and have nothing to add here, write "See above."*
Recruitment	*Describe how participants were recruited. Outline any potential self-selection bias or other biases that may be present and how these are likely to impact results.*
Ethics oversight	*Identify the organization(s) that approved the study protocol.*

Note that full information on the approval of the study protocol must also be provided in the manuscript.

Field-specific reporting

Please select the one below that is the best fit for your research. If you are not sure, read the appropriate sections before making your selection.

☒ Life sciences ☐ Behavioural & social sciences ☐ Ecological, evolutionary & environmental sciences

For a reference copy of the document with all sections, see nature.com/documents/nr-reporting-summary-flat.pdf

Life sciences study design

All studies must disclose on these points even when the disclosure is negative.

Sample size	For all imaging & scRNA-seq experiments we aimed for a minimum sample size of n=5 in order to have appropriate power given variability of the His Purkinje network and in the LAD surgery procedure, but mindful for the n3R requirements under our UK home office animal license; and for the same reason we performed cellPlex and demultiplexed our single cell RNA-seq data to identify individual heart of origin and ensure no anomalies were evident. Actual sample sizes used depended on litter size and survival rate following MI surgery in order to act in accordance with ethical requirements under our animal license; experiments were repeated with a new litter when there were fewer than 3 independent biological replicates surviving post-surgey. For scRNA-seq and ECG experiments to ensure appropriate controls were in place we used sibling pups to minimise genetic differences and were therefore most constrained by litter size and survival rate here.
Data exclusions	*Describe any data exclusions. If no data were excluded from the analyses, state so OR if data were excluded, describe the exclusions and the rationale behind them, indicating whether exclusion criteria were pre-established.*
Replication	The main imaging findings were replicated on independent days & with independent litters. The same results were found consistently across repeats. Experiments were repeated 3 independent times as indicated in the relevant figure legends throughout the manuscript. Attempts at

	replication were successful in all cases where animals survived through to analysis stages.
Randomization	Surgeon & experimenters were blinded to genotype where relevant (e.g., in case of nxk2-5/cre study); blinding in all analysis & wherever possible re treatment (MI vs sham - not possible on dissection because the suture know is visible on opening the rib cage but managed at all other times). Experimental groups were allocated randomly between MI versus control.
Blinding	Yes - blinding was performed.

Reporting for specific materials, systems and methods

We require information from authors about some types of materials, experimental systems and methods used in many studies. Here, indicate whether each material, system or method listed is relevant to your study. If you are not sure if a list item applies to your research, read the appropriate section before selecting a response.

Materials & experimental systems

n/a	Involved in the study
☐	☒ Antibodies
☒	☐ Eukaryotic cell lines
☒	☐ Palaeontology and archaeology
☐	☒ Animals and other organisms
☒	☐ Clinical data
☒	☐ Dual use research of concern
☒	☐ Plants

Methods

n/a	Involved in the study
☒	☐ ChIP-seq
☒	☐ Flow cytometry
☒	☐ MRI-based neuroimaging

Antibodies

Antibodies used	Chick GFP: Abcam ab13970 (1:200 dilution), Rabbit HCN4: Alamone Labs APC-052 (1:200 dilution), Goat CNTN2: R&D Systems AF4439 (1:100 dilution), Goat anti-chick-488: Invitrogen A32931 (1:200 dilution), Donkey anti-Rabbit-555 Invitrogen A-31572 (1:200 dilution), Donkey anti-goat-555 Invitrogen A-21432 (1:200 dilution), Donkey anti-rabbit-647, Invitrogen A-31573 (1:200 dilution).
Validation	Full validations with appropriate controls were performed for antibodies where relevant. Please see details on manufacturers websites (Abcam, Alamone labs, R&D systems, Invitrogen respectively). ab13790 is a chicken polyclonal antibody that is validated in GFP western blotting and immunofluorescence (tried and trusted by researchers since 2004, cited in over 3820 publications). Alamone APC-052 is knock-out validated highly specific antibody designed to recognise HCN4 from human, rat and mouse samples. R&D systems AF4439 has been validated by the company in mouse embryos with high specificity. Invitrogen antibodies have undergone advanced verification with additional specificity tests to ensure high confidence levels in these antibodies.

Animals and other research organisms

Policy information about studies involving animals; ARRIVE guidelines recommended for reporting animal research, and Sex and Gender in Research

Laboratory animals	Cx40EGFP/+, Nkx2.5Cre/+, Cx40-CreERT2 and Rosa26mTmG mouse strains used in this paper have been described previously and full references are provided in the manuscript. CD1 and C57BL/6 mice were obtained from Charles River Laboratories. Mice were housed by the Oxford Biomedical Services, with 12 hr light/dark cycles, controlled humidity and constant ambient temperature. All animals used in this study were less than 1 year old. Ages of neonatal mice used in experiments ranged from P0 - P21 as detailed individually for every relevant experiment.
Wild animals	No wild animals were used in this study.
Reporting on sex	Both sexes used but data not collected given the neonatal focus of this study
Field-collected samples	No field-collected samples were used in this study.
Ethics oversight	All experiments were carried out in accordance with the UK home office; project licenses PDDE89C84 and PP3194787, and were compliant with the UK Animals (Scientific Procedures) Act 1986.

Note that full information on the approval of the study protocol must also be provided in the manuscript.

Plants

Seed stocks	*Report on the source of all seed stocks or other plant material used. If applicable, state the seed stock centre and catalogue number. If plant specimens were collected from the field, describe the collection location, date and sampling procedures.*
Novel plant genotypes	*Describe the methods by which all novel plant genotypes were produced. This includes those generated by transgenic approaches, gene editing, chemical/radiation-based mutagenesis and hybridization. For transgenic lines, describe the transformation method, the number of independent lines analyzed and the generation upon which experiments were performed. For gene-edited lines, describe the editor used, the endogenous sequence targeted for editing, the targeting guide RNA sequence (if applicable) and how the editor was applied.*
Authentication	*Describe any authentication procedures for each seed stock used or novel genotype generated. Describe any experiments used to assess the effect of a mutation and, where applicable, how potential secondary effects (e.g. second site T-DNA insertions, mosiacism, off-target gene editing) were examined.*
